# The Translational Landscape of SARS-CoV-2-infected Cells Reveals Suppression of Innate Immune Genes

**DOI:** 10.1128/mbio.00815-22

**Published:** 2022-05-23

**Authors:** Maritza Puray-Chavez, Nakyung Lee, Kasyap Tenneti, Yiqing Wang, Hung R. Vuong, Yating Liu, Amjad Horani, Tao Huang, Sean P. Gunsten, James B. Case, Wei Yang, Michael S. Diamond, Steven L. Brody, Joseph Dougherty, Sebla B. Kutluay

**Affiliations:** a Department of Molecular Microbiology, Washington University School of Medicine, St. Louis, Missouri, USA; b Department of Genetics, Washington University School of Medicine, St. Louis, Missouri, USA; c Department of Pediatrics, Allergy, Immunology and Pulmonary Medicine, Washington University School of Medicine, St. Louis, Missouri, USA; d Department of Medicine, Pulmonary and Critical Care Medicine, Washington University School of Medicine, St. Louis, Missouri, USA; e Department of Medicine, Infectious Disease Division, Washington University School of Medicine, St. Louis, Missouri, USA; f Department of Pathology & Immunology, Washington University School of Medicine, St. Louis, Missouri, USA; g Department of Psychiatry, Washington University School of Medicine, St. Louis, Missouri, USA; University of Pennsylvania

**Keywords:** SARS-CoV-2, ribosome profiling, ribo-seq, mRNA translation, virus replication, ribosomal frameshifting, virus-host interaction, immune response, translational repression, programmed frameshifting

## Abstract

Severe acute respiratory syndrome coronavirus 2 (SARS-CoV-2) utilizes a number of strategies to modulate viral and host mRNA translation. Here, we used ribosome profiling in SARS-CoV-2-infected model cell lines and primary airway cells grown at an air-liquid interface to gain a deeper understanding of the translationally regulated events in response to virus replication. We found that SARS-CoV-2 mRNAs dominate the cellular mRNA pool but are not more efficiently translated than cellular mRNAs. SARS-CoV-2 utilized a highly efficient ribosomal frameshifting strategy despite notable accumulation of ribosomes within the slippery sequence on the frameshifting element. In a highly permissive cell line model, although SARS-CoV-2 infection induced the transcriptional upregulation of numerous chemokine, cytokine, and interferon-stimulated genes, many of these mRNAs were not translated efficiently. The impact of SARS-CoV-2 on host mRNA translation was more subtle in primary cells, with marked transcriptional and translational upregulation of inflammatory and innate immune responses and downregulation of processes involved in ciliated cell function. Together, these data reveal the key role of mRNA translation in SARS-CoV-2 replication and highlight unique mechanisms for therapeutic development.

## INTRODUCTION

The coronavirus (CoV) group encompasses a number of single-stranded, positive-sense RNA viruses with unusually large genomes (27 to 32 kb), which infect a wide range of animal species, including humans (1, 2). Presently, severe acute respiratory syndrome coronavirus 2 (SARS-CoV-2), the causative agent of the ongoing coronavirus disease 2019 (COVID-19) pandemic, continues to spread around the globe in part due to the emergence of viral variants with enhanced ability to be transmitted. Despite the development of highly effective vaccines that substantially reduced COVID-19-associated mortality, there are limited options for antiviral or immunomodulatory treatment for SARS-CoV-2. A basic understanding of the replicative mechanisms of SARS-CoV-2 and associated host responses in relevant settings can foster the development of virus-specific therapies.

SARS-CoV-2-induced lung disease is thought to be due in part to manipulation of host type I interferon (IFN) signaling (3). Compared to other CoVs, such as SARS-CoV and Middle East respiratory syndrome (MERS) virus, SARS-CoV-2 induces a poor or delayed IFN response in various experimental settings and *in vivo* (4–6). Extensive characterization of SARS-CoV-2-encoded proteins within the past 2 years has revealed multiple ways in which SARS-CoV-2 can translationally manipulate host gene expression and induction of innate immune responses. For example, NSP1 can bind to the mRNA entry channel of the 40S ribosomal subunit as well as nontranslating 80S ribosomes to prevent binding of capped mRNA and thus inhibit the formation of the translation initiation complex (7–10). Recent findings also implicate NSP14 as a translation inhibitor through its exoribonuclease and N7-methyltransferase activities (11). Under such inhibitory conditions SARS-CoV-2 mRNAs are thought to be efficiently translated owing to the structured elements within the 5′ untranslated regions (UTRs) of viral mRNAs (8, 12, 13). On the other hand, the bulk of published research on SARS-CoV-2-host interactions has relied on transcriptional profiling to study the immune response to infection (4, 14–18). Such approaches may not fully capture the host immune response to infection, in the face of viral mechanisms that block host mRNA translation. To address this shortcoming, similar to the work presented in our study, others have conducted ribosome profiling assays in SARS-CoV-2 infected Calu-3 lung cells and found that translation of newly transcribed mRNAs, including those that code for innate immune factors, can be translationally suppressed upon infection (13, 19, 20). Whether these observations can be recapitulated in other infection models, including the more physiologically relevant primary airway epithelial cells, remains unknown.

In addition to manipulation of host mRNA translation, SARS-CoV-2 utilizes programmed ribosomal frameshifting to successfully launch infection. The first two-thirds of the 5′ end of the SARS-CoV-2 genome is composed of two overlapping open reading frames (ORFs), ORF1a and ORF1b, which encode two polyproteins, pp1a and pp1ab (21). pp1a is produced when translation of the genomic RNA terminates at the stop codon of ORF1a. pp1ab is generated via a programmed −1 ribosomal frameshift (PRF) that occurs at the overlap between ORF1a and ORF1b, permitting the elongating ribosomes to bypass the termination signal in ORF1a (22). Many proteins encoded in ORF1b are part of the replication complex, thus making the −1 PRF to generate pp1ab a critical translational event for SARS-CoV-2 replication.

Frameshifting in CoVs is regulated by a highly conserved heptanucleotide slippery sequence (UUUAAAC) and an RNA pseudoknot structure a few nucleotides downstream (22). The current models of PRF suggest that ribosomes stall upon encountering the pseudoknot (23, 24). This event presumably enhances the efficiency of ribosomal frameshifting by forcing the ribosomes to pause on the slippery sequence, which in turn promotes the −1 slippage. CoV frameshifting is thought to occur at a high efficiency, with >50% of the ribosomes continuing into ORF1b (13, 20, 25), compared with other viruses such as HIV-1, in which only 5 to 10% of ribosomes move past the frameshifting element (26–28). Recent structural probing studies have revealed that alternative RNA conformations of the frameshifting element (FSE) may underlie the relatively high efficiency of frameshifting for SARS-CoV-2 (29). On the other hand, the behavior of ribosomes on SARS-CoV-2 RNAs, within the SARS-CoV-2 FSE, and throughout the course of infection in physiologically relevant settings has not been thoroughly studied.

Here, we conducted in-depth ribosome profiling studies to gain insight into the role of translational regulation in SARS-CoV-2 replication and the resulting host responses in the highly permissive Vero E6 epithelial cells and the more physiologically relevant primary human bronchial epithelial cells (HBECs) grown at an air-liquid interface (ALI). We found that SARS-CoV-2 mRNAs quickly dominated the cellular mRNA pool but were not translated at a higher efficiency than cellular mRNAs overall. In addition, ribosomes engaged with novel translation initiation sites (TIS) and accumulated within the slippery site. Nevertheless, ribosome occupancy downstream of the FSE was high, demonstrating the high efficiency of SARS-CoV-2 frameshifting. As for host responses, while numerous inflammatory chemokine genes, cytokine genes, and interferon-stimulated genes (ISGs) were upregulated transcriptionally in response to SARS-CoV-2 in the highly permissive Vero E6 cells, we found that many were not efficiently translated. Though we found that mRNAs encoding certain immune defense mediators were also less efficiently translated in SARS-CoV-2-infected primary HBECs, repression of host mRNA translation in this physiologically relevant system was overall more modest. Taken together, our results define the translational landscape of SARS-CoV-2-infected cells, revealing key events that may promote viral replication and disarm host immune responses at the level of mRNA translation.

## RESULTS

### Ribosome profiling reveals key features of the SARS-CoV-2 translational program.

**FIG 1:**
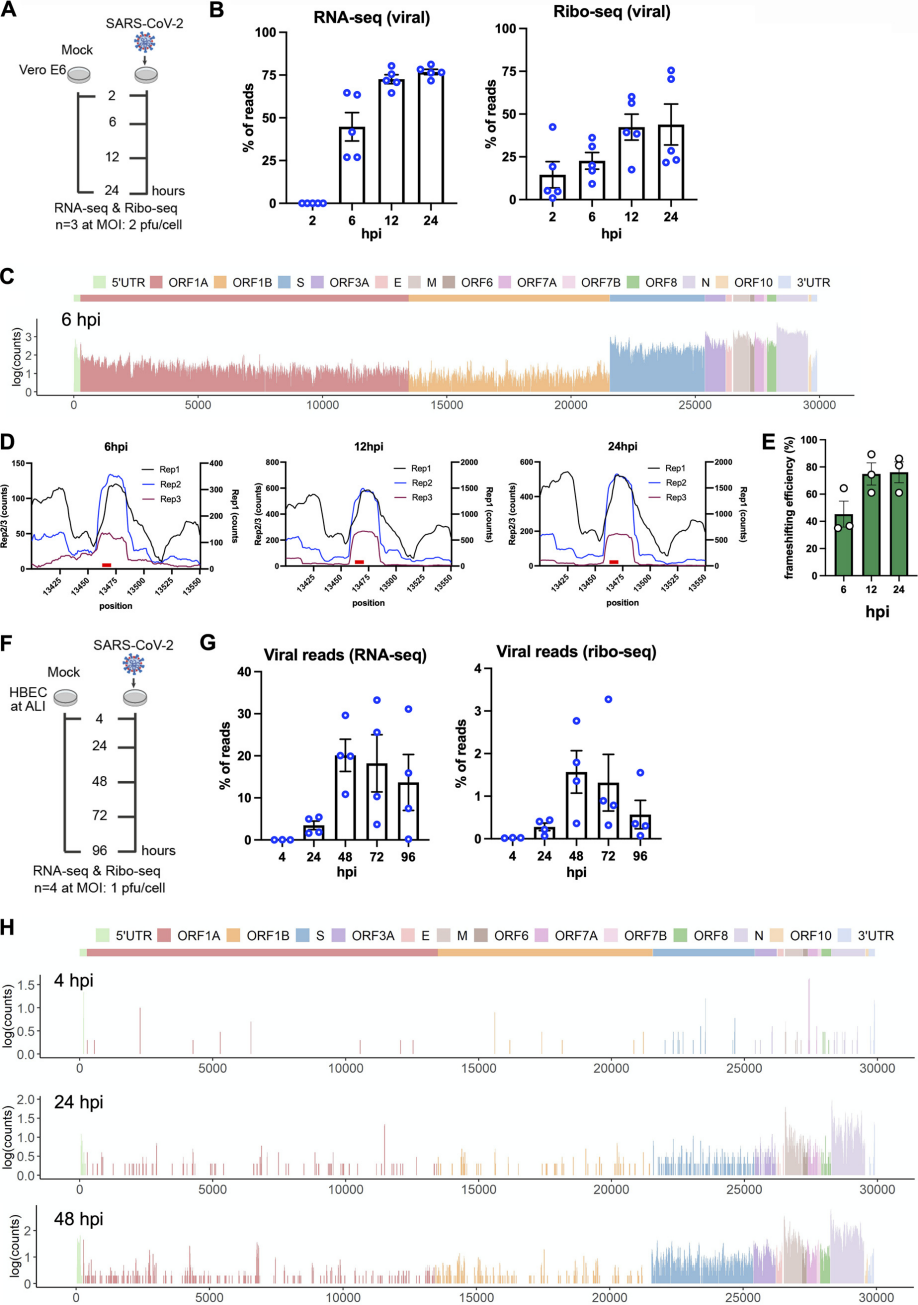
Ribo-seq reveals the translational program of SARS-CoV-2. (A) Schematic diagram of ribo-seq and RNA-seq experiments conducted in this study. Vero E6 cells were infected at 2 PFU/cell, and cells were processed for RNA-seq and ribo-seq at 2, 6, 12, and 24 hpi. (B) Percentages of RNA-seq and Ribo-seq reads uniquely mapping to SARS-CoV-2 and cellular transcripts at the indicated time points postinfection. Individual data points indicate independent biological replicates. (C) Ribo-seq counts (log_10_) along the viral genome at 6 hpi (see Fig. S3). The schematic diagram of SARS2 genome features shown at the top is colinear (see Table S3). (D) Ribo-seq read counts within the frameshifting site across three independent replicates at 6, 12, and 24 hpi. (E) SARS-CoV-2 frameshifting efficiency as determined by comparing the average read densities between ORF1a and ORF1b regions across three independent replicates and various time points postinfection. (F) Schematic diagram of ribo-seq and RNA-seq experiments conducted in this study. HBECs grown at ALI were infected at 1 PFU/cell and cells were processed for RNA-seq and Ribo-seq at 4, 24, 48, 72 and 96 hpi. (G) Percentage of RNA-seq and Ribo-seq reads uniquely mapping to SARS-CoV-2 and cellular transcripts at the indicated time points postinfection. Note that infection in this system progresses slower than in Vero E6 cells and a relatively small percentage of cells are infected at 24 and 48 hpi, as exemplified in Fig. S6. Individual data points indicate independent biological replicates. (H) Ribo-seq counts (log_10_) along the viral genome across various time points. The schematic diagram of SARS2 genome features shown at the top is colinear (Fig. S7 and Table S10).

To study the relationship between transcriptionally and translationally regulated events at early and late phases of SARS-CoV-2 infection, Vero E6 cells infected at a multiplicity of infection (MOI) of 2 PFU/cell were monitored by transcriptome sequencing (RNA-seq) and ribosome profiling (ribo-seq) during the course of infection for 24 h (Fig. 1A). Viral antigen staining of infected cells revealed that the majority of the cells were infected by 12 h postinfection (hpi) (see Fig. S1A [https://doi.org/10.5281/zenodo.6382957]). Triplicate sequencing libraries (RNA-seq and ribo-seq) were generated, and the mapping statistics are detailed in Tables S1 and S2 (https://doi.org/10.5281/zenodo.6382957). The quality of each sample and ribo-seq library was assessed as follows. First, despite the high degree of infection, RNA integrity was unaffected (Fig. S1B [https://doi.org/10.5281/zenodo.6382957]), suggesting that selection of polyadenylated mRNAs for RNA-seq is unlikely to introduce a major 3′ bias. Second, the length of distribution of ribo-seq reads that mapped to cellular and viral transcriptomes were within the expected range of ribosome protected fragments (Fig. S2A [https://doi.org/10.5281/zenodo.6382957]) (30, 31). We noted that in one replicate experiment, read lengths tended to be longer, likely due to less extensive nuclease digestion (data not shown). This library was excluded from relevant analyses downstream. Third, irrespective of the differences in the average read-length distribution of independent experiments, the majority of ribo-seq reads mapped to coding sequences (CDS) and 5′ UTRs, with a clear reduction in the fraction of reads mapping to 3′ UTRs compared to RNA-seq experiments done in parallel (Fig. S2B [https://doi.org/10.5281/zenodo.6382957]). Finally, mapped ribosome-derived reads within the CDSs were enriched in fragments that align to the translated frame for cellular mRNAs (Fig. S2C and D [https://doi.org/10.5281/zenodo.6382957]).

At 2 hpi, only a small fraction of cell-associated mRNA pool was derived from SARS-CoV-2 RNAs (Fig. 1B). At 6 hpi, a dramatic increase in viral RNA (vRNA) levels was observed, and by 12 hpi, nearly 80% of the total mRNA pool was of viral origin (Fig. 1B). Viral RNAs were present abundantly in the ribosome-bound pool as well, and by 12 hpi, ~50% of the ribosome-protected fragments contained SARS-CoV-2 sequences (Fig. 1B). Plotting of RNA-seq reads on the SARS-CoV-2 genome demonstrated that N-derived subgenomic RNAs (sgRNAs) were highly abundant throughout infection (Fig. S3A and Table S3 [https://doi.org/10.5281/zenodo.6382957]), a finding consistent with previous RNA-seq studies (32, 33). Ribosome density on SARS-CoV-2 mRNAs mirrored RNA abundance, with ribosomes enriched primarily on N-coding mRNAs by 6 hpi (Fig. 1C; Fig. S3B and Table S3 [https://doi.org/10.5281/zenodo.6382957]). Ribosome occupancy across viral RNAs increased further by 12 hpi and remained high during the remainder of infection (Fig. S3B). Ribosome footprints were nonuniform, with numerous high- and low-frequency binding sites observed reproducibly across viral RNAs (Fig. 1C; Fig. S3B) and with expected higher ribosome density within viral translation initiation sites (Fig. S3B). Overall, the translational efficiency of viral mRNAs was not substantially different from that of the majority of cellular mRNAs, with ORF1ab, S, and E mRNAs translated at a modestly higher efficiency and the remainder of viral mRNAs translated at a lower efficiency than average, a pattern that did not vary with progression of infection (Fig. S4 [https://doi.org/10.5281/zenodo.6382957]). Thus, the high abundance of viral mRNAs, as opposed to a specific regulatory mechanism, appears to ensure viral mRNA translation and protein abundance, a finding consistent with other published studies (13, 20).

Similar to what occurs in other CoVs, SARS-CoV-2 frameshifting is thought to be mediated by a conserved heptanucleotide slippery sequence (UUUAAAC) and an RNA pseudoknot downstream from it spanning nucleotides 13408 to 13540 (Fig. S5A [https://doi.org/10.5281/zenodo.6382957]). A notable local increase in ribosome occupancy was observed surrounding the slippery site within the frameshifting element (Fig. 1D; Fig. S5B and Table S3 [https://doi.org/10.5281/zenodo.6382957]), suggesting the possibility of steric hindrance by the FSE on translating ribosomes. Frameshifting was also evident in P-site analysis of the mapped reads, with a notable shift from frame 0 to frame 2 (−1 frame), before and after the frameshifting site (Fig. S5B). Comparison of read density distribution between ORF1a and ORF1b indicated a relatively high efficiency of frameshifting ranging from 50% to 75% throughout the course of infection (Fig. 1E), in line with published reports for SARS-CoV-2 as well as other CoVs (20, 25, 34).

SARS-CoV-2 primarily infects ciliated and type 2 pneumocyte cells in the human lung (35). Differentiated primary HBECs grown at the ALI represent one of the most physiologically relevant models to study SARS-CoV-2 infection in culture. To corroborate the above findings from Vero E6 cells, we performed similar ribo-seq studies in SARS-CoV-2-infected primary HBECs. Cells inoculated at an MOI of 1 PFU/cell were processed for RNA-seq and ribo-seq at 4, 24, 48, 72, and 96 hpi (Fig. 1F). In contrast to the highly permissive Vero E6 cells, the progression of infection in HBECs was relatively slow, and a small percentage of the cells were infected by 4 and 24 hpi (data not shown). SARS-CoV-2 spread was visible by 48 hpi, and the great majority of ciliated cells expressing ACE2 were infected by 96 hpi (Fig. S6A and B [https://doi.org/10.5281/zenodo.6382957]). In agreement, the amount of newly synthesized viral RNAs was low at 4 hpi, but by 48 hpi, approximately 20% of reads were of viral origin, and this value did not increase further at 72 and 96 hpi (Fig. 1G; Tables S4 and S5 [https://doi.org/10.5281/zenodo.6382957]). Of the relatively small number of RNA-seq reads that mapped to the viral RNAs at 4 hpi, the majority were derived from subgenomic viral mRNAs coding for N and to a lesser extent from upstream ORFs, including M, ORF6, ORF7, and ORF8 (Fig. S7A and Table S6 [https://doi.org/10.5281/zenodo.6382957]). Subgenomic viral mRNAs coding for N remained as the predominant species at later time points with notable increases at the expression level of upstream genes (Fig. S7A and Table S6).

Quality of HBEC-derived ribo-seq libraries was assessed as follows. First, RNA integrity was high despite widespread infection at 96 hpi (Fig. S8A [https://doi.org/10.5281/zenodo.6382957]). Second, length distribution of ribo-seq reads mapping to cellular and viral mRNAs matched the size expected from ribosome-protected fragments (Fig. S8B [https://doi.org/10.5281/zenodo.6382957]). Third, reads mapping to the 3′ UTRs were depleted in ribo-seq libraries (Fig. S9A [https://doi.org/10.5281/zenodo.6382957]). Fourth, ribo-seq libraries were enriched in fragments that align to the translated frame and had a dominant frame with a 3-nucleotide (nt) periodicity across various read lengths for both cellular and virally mapping reads (Fig. S9B and S10 [https://doi.org/10.5281/zenodo.6382957]).

In contrast to Vero E6 cells, viral RNAs constituted only a small fraction of ribo-seq-derived RNAs (Fig. 1G), suggesting a more restrictive translational environment overall for SARS-CoV-2 in primary HBEC-ALI cultures. Viral RNAs bound by host ribosomes were readily detected at 24, 48, and 72 hpi, but not at 4 hpi, with N and M ORFs being the most frequently translated (Fig. 1H; Fig. S7B [https://doi.org/10.5281/zenodo.6382957]). Overall translation efficiency of SARS-CoV-2 mRNAs was by and large proportional to the abundance of sgRNAs and proceeded in a similar cascade in the primary HBECs as well as in the Vero E6 cells. Due to the relatively low read coverage across ORF1ab, we did not further assess frameshifting efficiency in this experimental setting.

We next tested whether SARS-CoV-2 can utilize alternative translation initiation, which is increasingly recognized as a key posttranscriptional regulatory mechanism (36, 37). To do so, ribo-seq experiments were performed in the presence of harringtonine, which results in the accumulation of ribosomes at translation initiation sites. In addition to enrichment of ribosomes at the canonical start codons, harringtonine treatment resulted in accumulation of ribosomes at possible alternative translation initiation sites during the course of infection, albeit at generally lower frequencies. For example, at 6 hpi, an internal noncanonical start codon, UUG, within ORF M was utilized ~30% of the time and is predicted to result in an out-of-frame peptide of 53 amino acids (Tables S3 and S7 [https://doi.org/10.5281/zenodo.6382957]). An alternative translation initiation codon, AGG, at nt 21868 appeared to be utilized within S at 6, 12 and 24 hpi, which would result in a short (18-amino-acid) peptide (Tables S3 and S7). Finally alternative translation initiation sites were observed within M, resulting in an out-of-frame peptide and a truncated version of M (Tables S3 and S7).

### Inflammatory and innate immune mRNAs are inefficiently translated in SARS-CoV-2-infected cells.

Parallel analysis of ribo-seq and RNA-seq data sets provides a powerful tool to analyze translational level changes in response to SARS-CoV-2 infection. Paired RNA-seq and ribo-seq data obtained from three independent experiments were analyzed for differential gene expression patterns in Vero E6 cells. Principal-component analysis (PCA) showed that despite a degree of variation between replicates (likely due to the difficulty of synchronizing infections in the highly permissive Vero E6 cells), infected samples at later time points in infection clustered together (Fig. S11A [https://doi.org/10.5281/zenodo.6382957]) and that the biological coefficient of variation was within an acceptable range to detect significant changes in gene expression (Fig. S11B and C [https://doi.org/10.5281/zenodo.6382957]).

**FIG 2:**
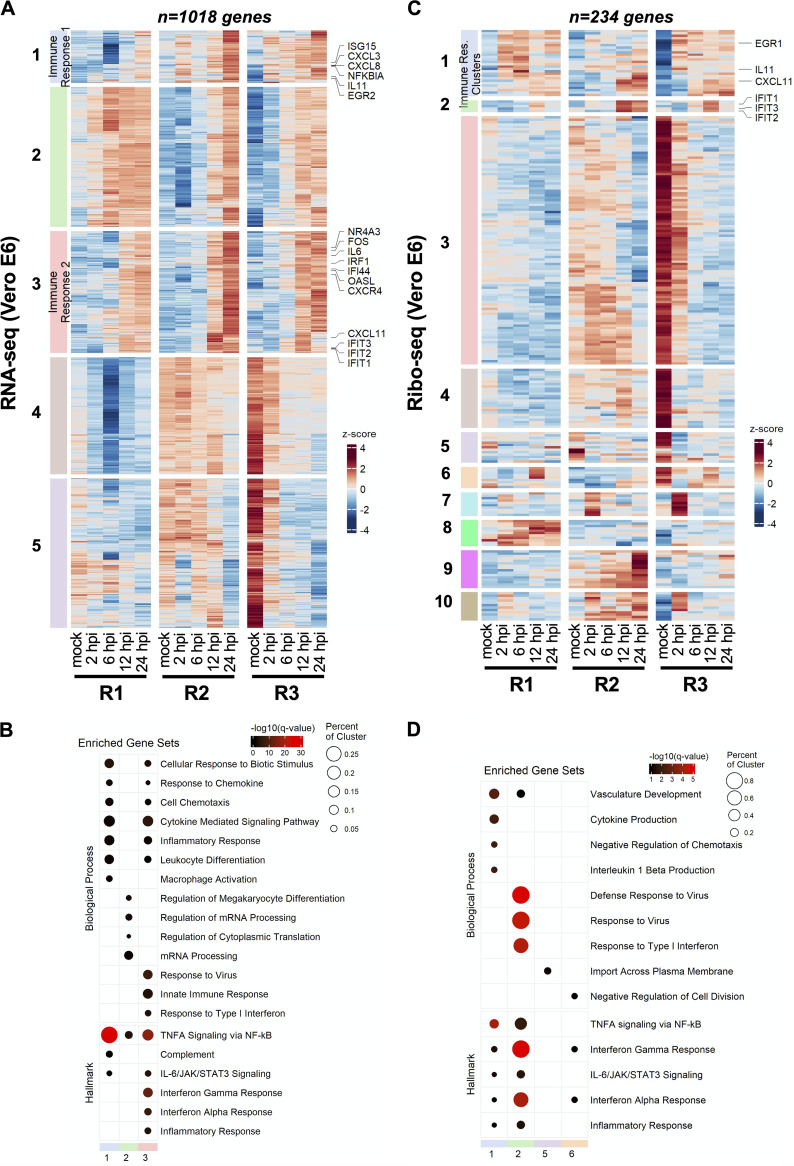
SARS-CoV-2 infection induces translational repression of innate immune genes. Vero E6 cells infected at 2 PFU/cell as detailed in Fig. 1 were analyzed for differential expression of host genes by RNA-seq (A and B) and ribo-seq (C and D). (A and C) Hierarchical clustering of DEGs after infection. Genes were filtered for an absolute log_2_ fold change of >2 and an adjusted *q* value of <0.05 at any time point. (B and D) Hypergeometric enrichment analysis from Hallmark and Gene Ontology databases for each individual cluster in 2A and 2C. Color represents significance (*q* value); size indicates the percentage of the cluster represented in the pathway. (See Tables S11 and S12.)

Hierarchical consensus clustering of the 1,018 differentially expressed genes (DEGs) (|log fold change [FC]| > 2 and false discovery rate [FDR] < 0.05) from RNA-seq generated 5 temporally resolved clusters (Fig. 2A; Fig. S12A and Table S8 [https://doi.org/10.5281/zenodo.6382957]). As early as 2 hpi, we found transcriptional upregulation of transcription factors involved in cell cycle regulation and induction of inflammation (i.e.NR4A3 and EGR3) (Fig. 2A; Fig. S13A and Table S8 [https://doi.org/10.5281/zenodo.6382957]). Numerous chemokine ligands (CXCL1, CXCL3, CXCL11, cluster 1) as well as IFN-α/β signaling and downstream ISGs significantly increased at 6 and 12 hpi (cluster 3) (Fig. 2A; Fig. S12A and S13A and Table S8). Induction of inflammatory and innate immune pathways was confirmed by gene set enrichment analysis (GSEA) of genes at each time point (Fig. 2B; Table S8). Another cluster (cluster 2) of upregulated genes was composed of genes involved in mRNA processing and mRNA translation (Fig. 2A and B; Table S8). Though numerous genes in clusters 4 and 5 were downregulated in all replicate experiments, we did not observe the specific enrichment of a pathway in this set of DEGs.

Remarkably, the majority of these transcript level changes were not apparent in the ribo-seq data (Fig. 2C and D; Fig. S12B and S13B and Table S9 [https://doi.org/10.5281/zenodo.6382957]). Only 234 genes were found to be differentially regulated in response to SARS-CoV-2 infection, forming 10 temporally resolved clusters (Fig. 2C; Fig. S12B and Table S9). Many of these clusters were smaller and the degree of differential expression varied in clusters 7 to 10 between replicate experiments (Fig. 2C; Fig. S12B and Table S9). Notwithstanding, cluster 2, which was enriched for type I IFN response and virus defense pathways, was substantially smaller and consisted of only a few ISGs (i.e., IFIT1, IFIT2, IFIT3, and CXCL10) (Fig. 2C and D; Fig. S12B and S13B and Table S9). Few other immune regulators, such as EGR1, interleukin 11 (IL-11), and CXCL11, were present in cluster 1 due to their temporal expression pattern, though innate immune pathways were not as prominently enriched in this cluster (Fig. 2C and D; Fig. S12B and S13B and Table S9). In contrast, we found that another innate immune modulator, IL-11, was significantly upregulated translationally but not transcriptionally at 2 hpi (Fig. S13B and Table S9). Clusters 3 and 4 consisted of genes that were downregulated significantly but were not enriched for a particular pathway (Table S9). Together, these findings suggest that immune response genes are translationally repressed and their expression is significantly delayed in infected cells.

Many of these findings were consistent for the RNA-seq and ribo-seq experiments performed on Vero E6 cells infected at a low MOI (Tables S10 and S11 [https://doi.org/10.5281/zenodo.6382957]). For example, transcription factors ATF3 and EGR1, key regulators of inflammatory responses, were upregulated at 24 hpi along with numerous chemokine ligands (i.e., CXCL1, CXCL8, and CXCL10) and interleukin 6 (Fig. S14A and Table S11 [https://doi.org/10.5281/zenodo.6382957]). We also noted the upregulation of numerous ISGs (i.e., IFIT1, IFIT2, IFIT3) as well as IFN-λ at 24 hpi (Fig. S14A and Table S11). The 48-hpi time point was marked by upregulation of genes involved in cell cycle regulation and apoptosis (i.e., FOS and NR4A3), as well as genes for inflammatory cytokines such as IL-31 and ISGs, including OASL (Fig. S14B and Table S11 [https://doi.org/10.5281/zenodo.6382957]). In line with our above observations, the great majority of the transcriptionally upregulated genes were not translationally upregulated at 24 hpi (Fig. S14B and Table S11) and 48 hpi (Fig. S14B and Table S11). The IL-11 and IL-1A genes stood out as immune-related genes that were translationally upregulated at 24 and 48 hpi, respectively (Fig. S14B and Table S11).

**FIG 3:**
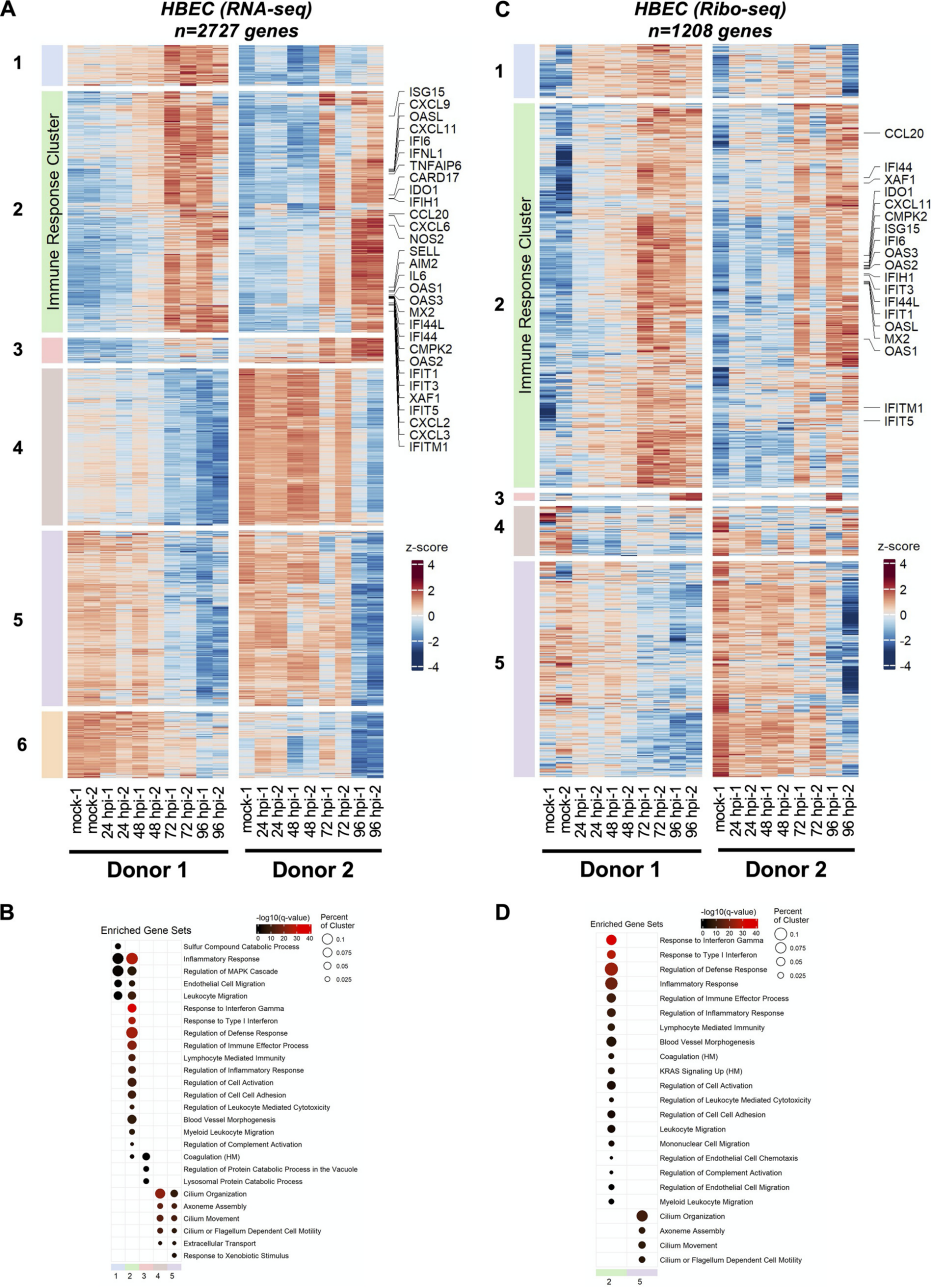
SARS-CoV-2-induced changes in primary airway cells. Primary human bronchial epithelial cells grown at an air-liquid interface were infected at 1 PFU/cell as detailed in Materials and Methods were analyzed for differential expression of host genes by RNA-seq (A and B) and ribo-seq (C and D). (A and C) Hierarchical clustering of DEGs after infection. Genes were filtered for an absolute log_2_ fold change of >2 and an adjusted *q* value of <0.05 at any time point. (B and D) Hypergeometric enrichment analysis from Hallmark and Gene Ontology databases for each individual cluster in 3A and 3C. Color represents significance (*q* value); size indicates the percentage of the cluster represented in the pathway (see Tables S15 and S16).

Paired RNA-seq and ribo-seq data obtained from four independent infections of HBEC-ALI cultures (from two independent donors) were analyzed for differential gene expression similarly. PCA showed that samples separated well based on time postinfection as well as donor (Fig. S15A [https://doi.org/10.5281/zenodo.6382957]). Furthermore, the degree of gene-level biological variability was within a reasonable range for both RNA-seq and ribo-seq libraries (Fig. S15B and C [https://doi.org/10.5281/zenodo.6382957]). SARS-CoV-2 infection induced differential expression of 2,727 and 1,208 genes in RNA-seq and ribo-seq experiments, respectively (Tables S12 and S13 [https://doi.org/10.5281/zenodo.6382957]). As expected from the low level of infection at 4 hpi, relatively few genes were differentially regulated at this time point for both RNA-seq and ribo-seq data sets (Tables S12 and S13). Transcriptionally upregulated genes formed six temporally resolved clusters (Fig. 3A; Fig. S16A and S17A and Table S12 [https://doi.org/10.5281/zenodo.6382957]). Cluster 2, which contained the largest number of upregulated DEGs, was significantly enriched in genes in the type I/III IFN pathway and inflammatory responses (Fig. 3B; Table S12). Clusters 4 and 5 were composed of genes that were downregulated at later stages of infection (Fig. 3A; Fig. S16A and S17A and Table S12).

GSEA revealed that many of these genes are involved in cilium organization and movement (Fig. 3B; Table S12), demonstrating the impact of SARS-CoV-2-induced remodeling and/or killing of the ciliated cells in the airway cultures. In contrast to Vero E6 cells, the majority of these patterns were maintained in ribo-seq experiments. The 1,208 DEGs derived from the ribo-seq experiments formed 5 clusters, with clusters 1 to 3 consisting of translationally upregulated genes (Fig. 3C; Fig. S16B and S17B and Table S13 [https://doi.org/10.5281/zenodo.6382957]). While clusters 1 and 3 were not enriched in genes in a specific pathway, we found that cluster 2 was significantly enriched in genes in the IFN and inflammatory response pathways (Fig. 3D; Table S13). Similar to RNA-seq data, GSEA of downregulated DEGs in cluster 5 revealed enrichment of genes involved in cilium organization and motility (Fig. 3D; Table S13). Overall, there was a relatively low degree of overlap between differentially expressed genes (both RNA-seq and ribo-seq) in Vero E6 and primary HBEC models of SARS-CoV-2 infection (Fig. S18A and B [https://doi.org/10.5281/zenodo.6382957]). However, a number of genes involved in antiviral defense, inflammatory response, and IFN pathways (i.e., CXCL10, CXCL11, IFIT1, etc.) were commonly upregulated in infected Vero E6 cells and HBECs at later stages of infection.

### Comparison of SARS-CoV-2 and host mRNA translation efficiencies.

**FIG 4:**
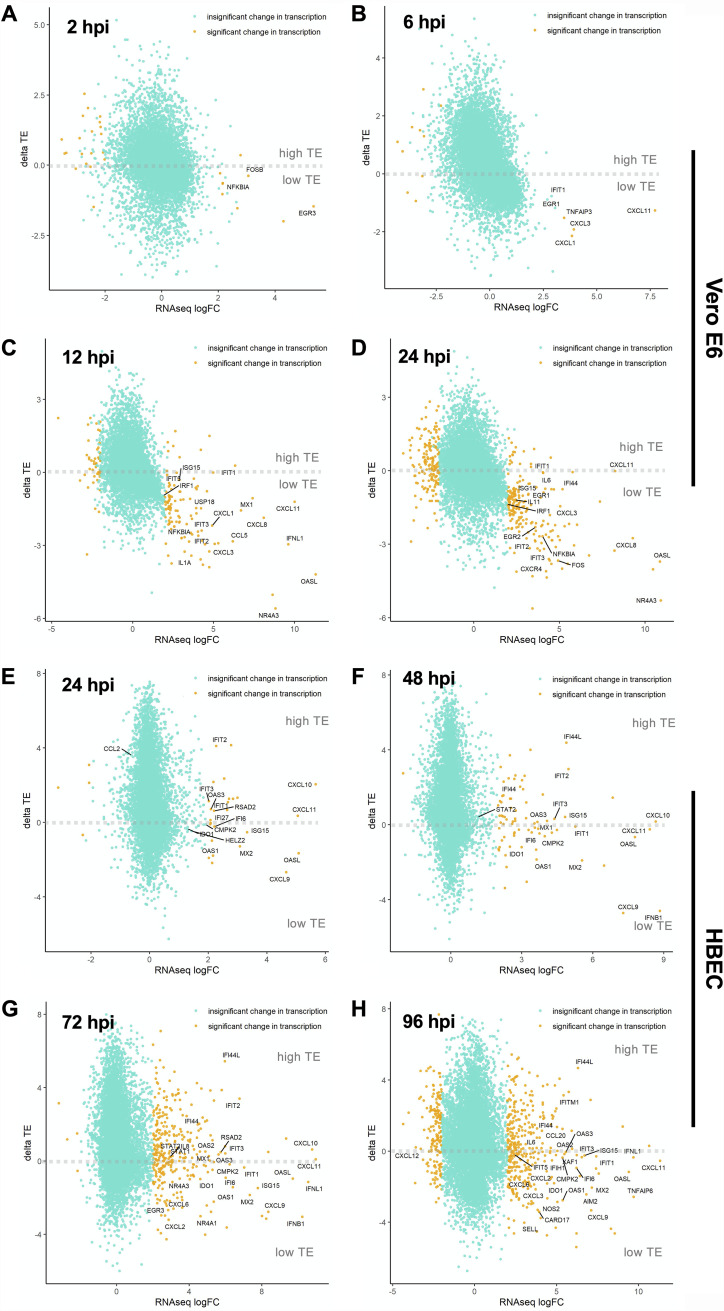
SARS-CoV-2-induces translational repression of innate immune genes. Changes in the translational efficiency of genes that were differentially transcribed in response to SARS-CoV-2 infection are shown for Vero E6 cells (A to D) and HBECs (E to H) at the indicated time points postinfection (see Tables S17 and S18).

We next compared the translational efficiency of cellular host response genes in Vero E6 and primary HBEC cultures. In Vero E6 cells, the translation efficiency of various immune modulatory genes was substantially lower than that of other cellular mRNAs, most evident at 12 and 24 hpi, which marks the accumulation of viral proteins (Fig. 4A to D; Table S14 [https://doi.org/10.5281/zenodo.6382957]). In contrast, in HBEC-ALI cultures, the translation efficiency of mRNAs encoding ISGs and inflammatory genes did not appear to be significantly lower than that of other cellular mRNAs (Fig. 4E to H; Table S15 [https://doi.org/10.5281/zenodo.6382957]). Notable exceptions included CXCL9 and IFN-B, which were substantially upregulated at 48 hpi at the transcript level but had comparably lower translation efficiencies (Fig. 4F; Table S15).

Comparative analysis of translational efficiency (TE) changes for immune-related genes in Vero E6 and primary HBECs revealed marked differences between the two models of SARS-CoV-2 infection. For example, at early times in infection (i.e., 2 hpi for Vero E6 cells and 24 hpi for HBECs), translation efficiencies of selected innate immune genes were high and correlated reasonably well between the two models (Fig. S19A [https://doi.org/10.5281/zenodo.6382957]). However, as infection progressed, suppression of innate immune response genes was particularly evident in the Vero E6 cells compared to primary HBECs (Fig. S19B to D [https://doi.org/10.5281/zenodo.6382957]).

**FIG 5:**
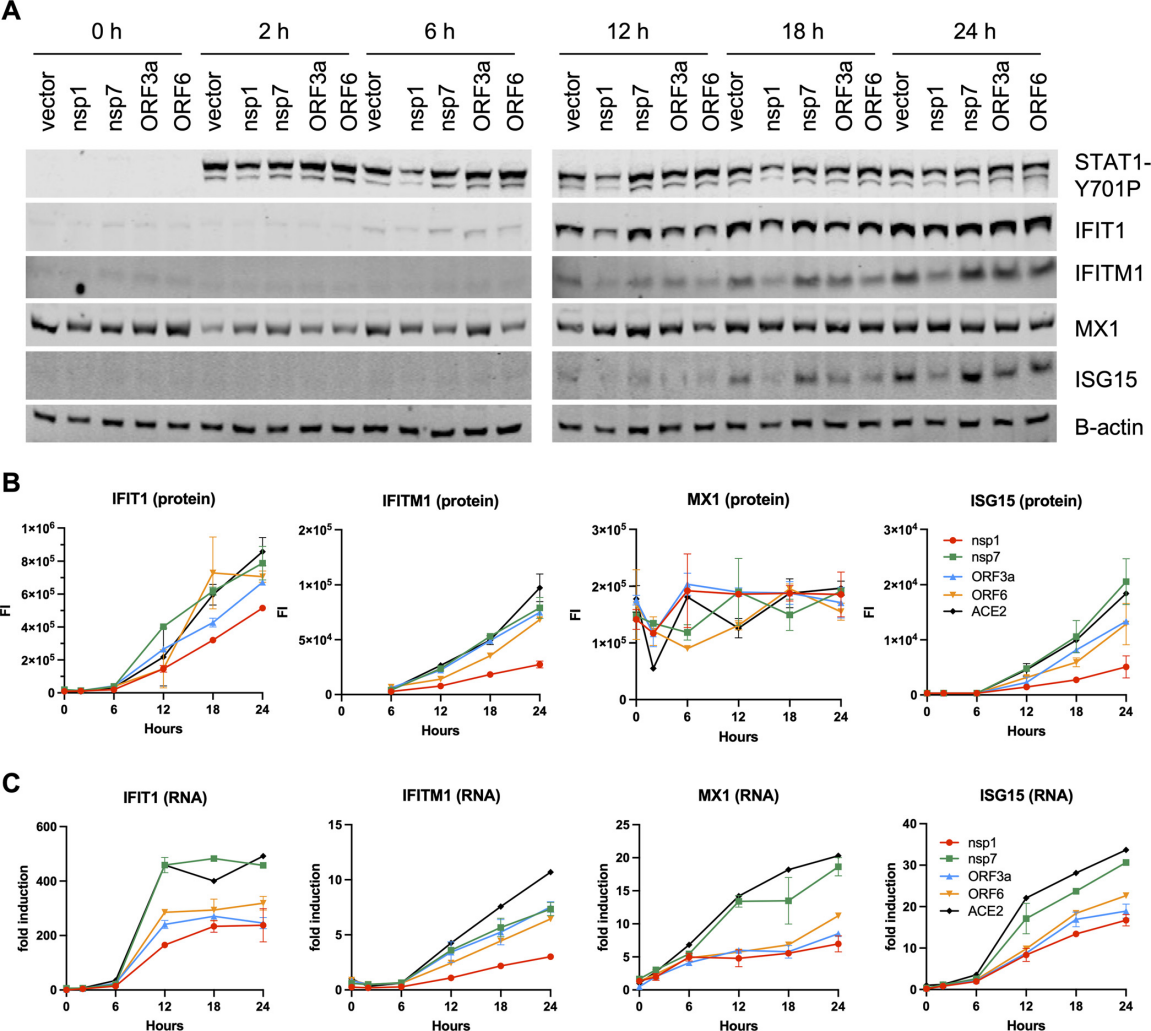
SARS-CoV-2 proteins block the type I IFN response at different stages. HEK293T cells were transfected with NSP1, NSP7, ORF3a, and ORF6 expression plasmids and treated with 1,000 U of IFN-α. Cells were analyzed for ISG induction by immunoblotting (A and B) and RT-qPCR (C). Data are derived from two independent experiments. Data in B, C show the mean and error bars represent the standard error.

Numerous viral proteins have been implied in modulation of type I IFN responses, and we next tested the direct impact of some of these factors in suppression of ISG expression. To this end, cells transfected with Nsp1, Nsp7, ORF3a, and ORF6 expression constructs were stimulated with IFN-α, and induction of ISGs was assessed by immunoblotting and reverse transcription-quantitative PCR (RT-qPCR). We found that Nsp1 overexpression significantly reduced IFN-α-mediated phosphorylation of STAT1 (Fig. 5A), whereas other viral proteins had no impact on STAT1 levels or phosphorylation. Nsp1-mediated suppression of STAT1 phosphorylation was accompanied by a significant reduction of ISG upregulation at both the protein and RNA levels (Fig. 5A to C). While ORF3a and ORF6 did not affect STAT1 phosphorylation, they both reduced steady-state ISG expression (Fig. 5A), yet the impact on ISG protein levels was relatively modest (Fig. 5B). In line with other published studies (38, 39), these findings suggested that the observed translational repression of innate immune modulators in SARS-CoV-2-infected cells is likely due to the actions of multiple viral proteins and possibly due to virus-induced changes and stress in heavily infected cells.

## DISCUSSION

Here, we utilized ribosome profiling (ribo-seq) coupled with RNA-seq to study the translational events that regulate viral gene expression and host responses over multiple time points after SARS-CoV-2 infection in different cell culture models. SARS-CoV-2 replicates rapidly, with viral RNAs constituting the great majority of the total mRNA pool soon after infection. Our data show that viral mRNA abundance is the main determinant of efficient viral mRNA translation and that SARS-CoV-2 mRNAs sequester ribosomes from the translating pool by competition, simply outnumbering the host counterparts. This observation notwithstanding, certain viral mRNAs (i.e., those encoding S, E, and ORF1ab) were translated modestly more efficiently than others. While the overall conclusions are similar, another study found that ORF1ab was less efficiently translated than other viral mRNAs (20), which we ascribe to possible differences in read depth (with our study having higher read depth within ORF1ab), RNA-seq approaches, infection conditions, and cell types.

We observed that SARS-CoV-2 employs a highly efficient frameshifting strategy to facilitate virus replication. In line with the model that ribosomes pause at the slippery sequence upon encountering the pseudoknot (40, 41) and recent structural studies of the ribosome-bound SARS-CoV-2 frameshifting element (42), we observed a local increase in ribosome density overlapping the slippery sequence for SARS-CoV-2 (Fig. 1D; Table S3). Nevertheless, ribosome density downstream of the frameshifting site within the SARS-CoV-2 ORF1b was high, with ribosomes continuing into the ORF1b frame >50% of the time. In addition to the sequence-specific differences within the slippery site between SARS-CoV-2 and other viruses that utilize frameshifting (43), structures downstream of the slippery sequence or alternative structural conformations of the FSE may underlie the high efficiency of SARS-CoV-2 frameshifting (22, 29).

In addition, antiviral host proteins can also affect viral mRNA translation and frameshifting. For example, an ISG product, known as C19orf66 (Shiftless), has been demonstrated to impair HIV-1 and Japanese encephalitic virus replication through inhibition of PRF (44, 45) and dengue virus replication through inhibition of viral translation (46). Furthermore, C19orf66 associates with host ribosomes, and it can inhibit PRF of different coronaviruses, including SARS-CoV-2 *in vitro* (47), and has been found to associate with SARS-CoV-2 RNAs in cells (48). Of note, we found that C19orf66 was upregulated approximately 5-fold at both the transcript and protein levels in HBECs, but not in Vero E6 cells (Fig. S20 [https://doi.org/10.5281/zenodo.6382957]), which may contribute to the overall more restrictive state of viral translation in the HBEC model.

Our study provides an in-depth picture of how host cell responses to SARS-CoV-2 are regulated at the transcriptional and posttranscriptional levels. In the highly permissive Vero E6 cells, we observed upregulation of proinflammatory chemokines as early as 6 hpi followed by a more delayed induction of ISGs, a finding in line with previous observations in immortalized lung cell line models of SARS-CoV-2 (4, 13, 19). However, the great majority of the innate immune response genes appeared to be translated at a low efficiency (Fig. 2A to D; Table S14), a finding also in line with other ribo-seq studies conducted on SARS-CoV-2-infected Calu-3 lung cells (13, 19). Apart from this specific effect on innate immune genes, we did not observe a global decrease in host mRNA translation *per se*, and most cellular mRNAs were translated proportionally to their mRNA abundance.

Translational repression of innate immune genes was less apparent in the complex setting of primary HBECs grown at the ALI, though several chemokine ligands and IFN-β tended to be less efficiently translated (Fig. 4E to H; Table S15). The potential factors that underlie these differences between Vero E6 cells and HBEC-ALI cultures are manifold. First, Vero E6 cells, as well as other cell line models broadly used in the field, overexpress ACE2 and are unusually permissive to infection, allowing quick accumulation of viral proteins with established effects on host mRNA degradation and translation. Second, the majority of published models for SARS-CoV-2 infection utilized cancer-derived cell lines (i.e., Calu-3, A549, Caco-2, and Huh7), which often lack key arms of innate immunity. In fact, it is apparent in the HBEC-ALI model that viral translation, and therefore accumulation of viral proteins, may overall be more restricted than in the highly permissive Vero E6 cells. Third, the HBEC-ALI model is composed of basal, club, and BC/club cells that do not express/express low levels of ACE2 and hence are not as efficiently infected by SARS-CoV-2 (though there is some evidence that SARS-CoV-2 can be detected in these cells at later stages of SARS-CoV-2 replication [49]). Thus, it is possible that the observed upregulation of inflammatory and innate immune genes takes place in the uninfected bystander cells that do not express viral proteins, a finding consistent with recent small cytoplasmic RNA sequencing (scRNA-seq) studies (49). Although some of these differences can in principle be circumvented by conducting HBEC infections at a higher MOI, the effective MOI is much lower in the HBECs, as is the case in most primary cell culture models for a broad number of viral pathogens. Furthermore, it is difficult to gauge the effective MOI in HBECs, as susceptibility to SARS-CoV-2 varies from donor to donor, the ALI interface limits the amount of virus inoculum that can be used (as ciliated cells need to remain in contact with air), and the natural production of mucus limits viral spread.

The apparently low translation efficiency of mRNAs involved in antiviral defenses in Vero E6 cells may be mediated by the SARS-CoV-2 protein NSP1, which associates tightly with ribosomes to prevent binding of capped mRNA and thus inhibit the formation of the translation initiation complex (7–10, 50). Given the high abundance of ribosomes in the cell, whether physiologically relevant concentrations of NSP1 are sufficient to induce a global block in mRNA translation is unclear. For example, even in cells overexpressing NSP1, we found no evidence of a translational block to ISG expression (Fig. 5). Rather, NSP1 expression blocked STAT1 phosphorylation and subsequently reduced transcriptional induction of ISGs. SARS-CoV-2 encodes numerous nonstructural and accessory proteins, some with functions in antagonism of IFN responses through inhibition of IFN production and IFN signaling in infected cells. For example NSP6, NSP13, ORF3a, M, N, ORF6, ORF7a, ORF7b, and ORF8 inhibit IFN signaling (3, 38, 39, 51–55), whereas NSP1, NSP5, NSP6, NSP15, ORF6, and ORF7b can block the production of IFN-β (54–56). Thus, the observed translational repression of ISGs in the heavily infected Vero E6 cells is likely due to a complex interplay between viral mRNAs dominating the cellular mRNA pool, viral accessory proteins such as NSP1, and possibly reduced translation initiation due to cellular stress induced by SARS-CoV-2. Finally, we cannot rule out the possibility that the translational suppression of innate immune genes is also contributed to by the host’s attempt to curb viral replication, including upregulated expression of members of the IFIT family with known functions in translation inhibition (57–61). Future studies are warranted to empirically test these possibilities and define the mechanism of apparent innate immune suppression.

It is well documented that SARS-CoV-2 can induce degradation of host mRNAs (13, 62) and inhibit nuclear export of cellular mRNAs (13, 63), which may explain the high abundance of the viral mRNAs as shown in Fig. 1B. Our analyses focused solely on the relative translation efficiency of cytosolic viral and host mRNAs (i.e., translation per mRNA molecule) but did not factor in the absolute abundance of host mRNAs or absolute protein abundance, which may introduce certain biases in analyses and is a limitation of our approach (64, 65).

Taken together, our results provide novel insights into and a rich resource on how translational regulation shapes SARS-CoV-2 replication and host responses. While COVID-19 pathogenesis is in part due to virus-induced destruction of infected cells, elevated production of inflammatory mediators and the virus-induced immunopathology are thought to play a big role in SARS-CoV-2-induced lung injury (66, 67). Our findings suggest that immune responses in actively infected cells may be dampened or delayed for SARS-CoV-2 to efficiently replicate and release viral progeny. As such, it is possible that the elevated levels of inflammatory mediators *in vivo* are due to bystander cells or infection of immune cell subsets, such as monocytes and macrophages, that are less permissive to SARS-CoV-2 but can sense and respond to infection by secretion of immune modulatory molecules (68). Altogether, our study provides an in-depth picture of translationally regulated events in SARS-CoV-2 replication and reveals that impairment of host mRNA translation may allow SARS-CoV-2 to evade host immunity. Modulation of viral RNA structures and proteins that regulate mRNA translation will provide a unique avenue for therapeutic development.

## MATERIALS AND METHODS

### Chemicals and reagents.

Standard laboratory chemicals were obtained from reputable suppliers such as Sigma-Aldrich. Cycloheximide (CHX) was obtained from Sigma, dissolved in ethanol and stored at −20°C. Harringtonine (HT) was purchased from LKT Laboratories, Inc., resuspended in dimethyl sulfoxide (DMSO), and stored in aliquots of 2 mg/mL at −20°C. IFN-α was purchased from PBL Assay Sciences and stored at −80°C per the manufacturer’s instructions.

### Plasmids and viruses.

Mammalian expression plasmids encoding SARS-CoV-2 genes (NSP1, NSP7, ORF3a, and ORF6) were obtained from BEI Resources and propagated as recommended. SARS-CoV-2 strain 2019-nCoV/USA-WA1/2020 was obtained from Centers for Disease Control and Prevention (a gift from Natalie Thornburg), and SARS-CoV-2 Neon-green (SARS-CoV-2-NG) reporter virus has been described before (69). SARS-CoV-2 was propagated in Vero CCL-81 cells (ATCC-CCL-81) or its derivative engineered to stably express human TMPRSS2 (obtained from the Whelan lab [70]) to minimize accumulation of spike mutations. For virus propagation, cells were infected at an MOI of 0.01 in Dulbecco’s modified Eagle medium (DMEM; Sigma) supplemented with 2% fetal bovine serum (FBS; VWR) and 10 mM HEPES buffer (Corning), and cell culture supernatants were collected after visible cytotoxicity was reached, at ~3 days postinfection. Virus stock titers were determined on Vero E6 cells (ATCC-CRL-1586) by plaque assays and sequenced to confirm identity to the reference sequence.

### Cells and infections.

All cell lines were maintained in a humidified incubator at 37°C with 5% CO_2_ unless otherwise indicated. HEK293T cells (ATCC CRL-11268) were cultured in Dulbecco’s modified Eagle medium supplemented with 10% fetal bovine serum. HEK293T cells grown in 24-well dishes were transfected with mammalian expression plasmids encoding SARS-CoV-2 genes (NSP1, NSP7, ORF3a, and ORF6) using polyethyleneimine (PolySciences, Warrington, PA).

Vero and Vero E6 cells (and derivatives thereof) were cultured in DMEM supplemented with 10% FBS and 10 mM HEPES (pH 7.4). For RNA-seq and ribo-seq experiments, Vero E6 cells grown in 6-well culture dishes were inoculated with SARS-CoV-2 strain 2019-nCoV/USA-WA1/2020 in DMEM supplemented with 2% FBS for an hour in a humidified incubator at 37°C, after which the initial inoculum was removed and replaced with cell culture medium.

Primary human bronchial epithelial cells (HBECs) grown at an air-liquid interface (ALI) were processed as follows. Human airway epithelial cells were isolated from surgical excess of tracheobronchial segments of lungs donated for transplantation as previously described and were exempt from regulation by U.S. Department of Health and Human Services regulation 45, Code of Federal Regulations, Part 46 (71). Tracheobronchial cells were expanded in culture, seeded on supported membranes (Transwell; Corning, Inc.), and differentiated using ALI conditions as detailed before using 24-well inserts (72, 73). Prior to infection, HBECs were washed two or three times with 1× phosphate-buffered saline (PBS) to remove the mucous layer, which otherwise can slow down infection. For ribo-seq/RNA-seq experiments, HBECs were inoculated with SARS-CoV-2 strain 2019-nCoV/USA-WA1/2020 as described above in DMEM supplemented with 2% FBS for 2 h in a humidified incubator at 37°C, after which the initial inoculum was removed. Cells were washed with 1× PBS to remove the virus inoculum and maintained at the ALI for the duration of the assays. For monitoring virus spread in HBECs, cells were infected with the SARS-CoV-2-NG reporter virus similarly and imaged by epifluorescence microscopy in a biosafety level 3 (BSL3) setting at time intervals indicated in the figure legends. In other experiments, HBECs infected with 2019-nCoV/USA-WA1/2020 were fixed and subjected to RNA *in situ* hybridization as detailed below.

### Immunofluorescence.

Infected Vero E6 cells and HBECs were fixed with 4% paraformaldehyde for 20 min at room temperature, followed by permeabilization using 0.5% Tween 20 in PBS for 10 min. Cells were blocked with 1% bovine serum albumin (BSA) and 10% FBS in 0.1% PBS–Tween 20 (PBST) for 1 h prior to staining with a rabbit polyclonal anti-SARS-CoV-2 nucleocapsid antibody (Sino Biological, Inc., catalog no.40588-T62) diluted 1:500 and incubated overnight at 4°C. The following day, cells were stained with an Alexa Fluor 488-conjugated goat anti-rabbit secondary antibody (Invitrogen) at a 1:1,000 dilution, counterstained with DAPI (4′,6-diamidino-2-phenylindole), and imaged by immunofluorescence microscopy.

### RNA *in situ* hybridization.

Primary human airway epithelial cells were fully differentiated at air-liquid interface on supported plastic membranes (Transwell; Corning) as detailed above. Cells were fixed by immersion of the Transwell membrane in methanol-acetone (50%:50% [by volume]) at −20°C for 20 min followed by 4% paraformaldehyde at room temperature for 15 min. Cells were washed three times with 1× PBS and stored at 4°C. Prior to probing for vRNA, membranes containing cells were cut from plastic supports, divided into 4 pieces, and placed in a fresh 48-well plate. RNA detection was performed using the manufacturer’s protocol for RNAscope fluorescent *in situ* hybridization (RNAscope multiplex fluorescent assay kit, v2; Advanced Cell Diagnostics). Briefly, cells on membranes were treated with 3% hydrogen peroxide for 10 min at room temperature, washed with distilled water, treated with protease III solution, and diluted 1:15 in 1× PBS for 10 min in a humidified hybridization oven at 40°C. The cells were then washed with PBS and incubated for 2 h at 40°C with manufacturer-designed antisense probes specific for the SARS-CoV-2 positive-strand S gene encoding the spike protein (RNAscope Probe-V-nCoV2019-S; catalog no. 848561) or ORF1ab (RNAscope Probe-V-nCoV2019-orf1ab-O2-sense-C2; catalog no. 854851-C2). The probes were visualized according to the manufacturers’ instructions by incubation with RNAscope amplifiers, horseradish peroxidase, and fluorescent label (Opal fluorophores; Perkin-Elmer). Membranes were mounted on glass slides using anti-fade medium containing DAPI (Fluoroshield; Sigma-Aldrich). Images were obtained using a 5000B Leica microscope equipped with a charge-coupled device camera (Retiga 200R) interfaced with QCapture Pro software (Q Imaging).

### Ribosome profiling.

Ribosome profiling (ribo-seq) was performed as described before with the following modifications (30, 31). Mock-, HIV-1-, and SARS-CoV-2-infected cells were treated with complete cell culture medium supplemented with 0.1 mg/mL CHX for 1 min at room temperature followed by one round of washing in ice-cold PBS supplemented with 0.1 mg/mL CHX. Cells were lysed in 1× mammalian polysome lysis buffer (20 mM Tris HCl [pH 7.4], 150 mM NaCl, 5 mM MgCl_2_, 1% Triton X-100, 0.1% NP-40, 1 mM dithiothreitol [DTT], 10 U of DNase I, with 0.1 mg/mL CHX). The cells were then triturated by repeated pipetting and incubated with lysis buffer for at least 20 min to ensure virus inactivation. Lysates were centrifuged for 10 min at ≥20,000 × *g* at 4°C for clarification. The supernatants were split into multiple aliquots, with SDS added to one aliquot to a final concentration of 1% for downstream RNA-seq sample preparation, and flash frozen in a 70% ethanol-dry ice bath or directly placed at −80°C. RNA extracted from lysates were subjected to Bioanalyzer RNA-Nano analysis. An RNA integrity number (RIN) of 8 and above (maximum RIN = 10) is considered to indicate intact RNA. Lysates were treated with RNase I (5 U/OD_260_ unit), and ribosome-protected fragments were isolated via centrifugation through Microspin S-400 HR columns (GE Healthcare) and purified using the RNA Clean and Concentrator kit (Zymo Research). Recovered ribosome-bound fragments (RBFs) were then subjected to rRNA depletion using RiboZero beads from the TruSeq Gold stranded total RNA library preparation kit (Illumina) and purified using a Zymo RNA Clean and Concentrator kit. Fragments were then end-labeled with [γ-^32^P]ATP using T4 polynucleotide kinase (New England Biolabs [NEB]), separated on 15% Tris-borate-EDTA (TBE)–urea gels, and visualized by autoradiography. RNA fragments of ~30 nt were excised from the gels and purified as detailed before in 400 μL of 0.4 M NaCl supplemented with 4 μL SUPERaseIN (Thermo Fisher). 3′ and 5′ adapters were sequentially ligated as in a previously described protocol (74, 75), reverse transcribed, and PCR amplified. We acknowledge that our ligation-based library generation protocol may introduce biases toward inserts containing distinct nucleotides at the 5′ and 3′ ends. Indeed, we found a modest preference toward Us and Cs in the first position and Gs and Cs in the last position of inserts. Libraries were then sequenced on HiSeq-2000 or NextSeq 500 platforms (Illumina) at the Genome Technology Access Center or the Edison Family Center for Genome Sciences & Systems Biology, respectively, at Washington University School of Medicine.

### RNA-seq.

An aliquot of cell lysates harvested from ribo-seq experiments above was processed in parallel for RNA-seq using a TruSeq stranded mRNA library preparation kit (Illumina) following extraction using a Zymo RNA Clean and Concentrator (5) kit. RNA-seq libraries were constructed using TruSeq RNA single-index adapters and deep sequenced as described above at Washington University in St. Louis, MO.

### Data analysis.

All of the data analysis pipelines used in this study are available at https://github.com/kutluaylab/SARS-2_COVID. Below we detail the salient steps of data analyses.

### (i) Mapping.

RNA-seq and ribo-seq data sets were analyzed by publicly available software and custom scripts. In brief, for ribo-seq, reads were separated based on unique barcodes and the adapters trimmed using BBDuk (http://jgi.doe.gov/data-and-tools/bb-tools/). The resulting reads were first mapped to rRNA to remove any rRNA-derived reads not completely removed by depletion kits during library generation. Reads were then sequentially mapped to the SARS-CoV-2 and host genomes. In brief, Bowtie aligner (76) was used for viral genome/transcriptome mapping (mapping criteria: -v 1, -m 10), and STAR aligner (77) was used for mapping (mapping criteria outFilterMismatchNoverLmax 0.04, outSAMstrandField intronMotif, outSAMattributes All, quantMode TranscriptomeSAM) of reads to the African green monkey (AGM) (Chlorocebus sabaeus) or human genome (hg19). For ribo-seq reads that map to the SARS-CoV-2 genome, reads were additionally collapsed to minimize PCR overamplification artifacts with the aid of UMI barcodes. For AGM/human alignments, mapped reads were annotated using the featureCounts package (78) and GTF annotation files freely available from NCBI and Ensembl.

### (ii) Read length distributions.

Ribo-seq read length distributions were generated from cellular transcriptome alignment and viral sorted alignment BAM files. Unmapped alignments were discarded with SAMtools (79), and the number of aligned reads of each length was counted and plotted.

### (iii) Genomic region counts.

The 5′ UTR, CDS, and 3′ UTR region counts were generated for each sample as follows. The counts of mock-infected and infected samples, separated by time point, were used as input. Protein coding genes were filtered such that they have >1 count per million (CPM) in at least half of the ribo-seq samples and half of the RNA-seq samples. Annotations of 5′ UTRs, CDS, and 3′ UTRs of these filtered genes were retrieved, and repetitive low-complexity elements were removed. The aligned BAM files were recounted using the regional annotation files with featureCounts with the following parameters: -t CDS/UTR5/UTR3 -g gene_id –fracOverlap 1 –minOverlap 1.

### (iv) PCA.

For the PCA, the ribo-seq and RNA-seq counts were first filtered by the filterByExpr function from the edgeR R package (80). In the normalization step, library sizes were adjusted using the trimmed-mean-of-M-values method as implemented in the calcNormFactors function, and read counts were converted to CPM. In a second filtering step, genes with more than 1 CPM in all samples were selected, and the filtered CPM values were log_10_ transformed. PCA was performed with the prcomp R function with data centering.

### (v) P-site and alternative TIS analysis.

The R package riboWaltz (81) and the Ribo-TISH package (82) were utilized to determine the location of ribosomal P sites with respect to the 5′ and 3′ end of reads, as well as to illustrate triplet periodicity and determine the percentage of reads within each frame in the CDS and UTR. Transcriptome alignment BAM, sorted alignment BAM, and corresponding cellular and viral annotations were used as input. An additional parameter used in the Ribo-TISH quality function was –th 0.4.

Alternative TIS sites in both host and viral reads were found using the Ribo-TISH package (82). For viral TIS, analysis was carried out in the “predict” mode comparing samples mock-treated or treated with harringtonine at each time point (with replicates). This was replicated for host analysis, although with the additional step of analysis in the “diff” mode to predict TIS differentially regulated between infected and uninfected cells.

### (vi) Differential gene expression analysis.

Differential gene expression analysis was carried out using the edgeR package (80) with read counts produced by featureCounts as described in the mapping section. Considering that virally derived sequences quickly dominated the host mRNA pool, for differential gene expression of host mRNAs, library sizes were normalized relative to reads that mapped only to host mRNAs. Library sizes were adjusted using the trimmed-mean-of-M-values method as implemented in the calcNormFactors function. Genes with >1 CPM in at least half of the samples were selected for the analysis. Common, trended, and tagwise dispersion was estimated with the estimateDisp function (robust = T). Genewise coefficient of variation was plotted against log_2_ CPM using the plotBCV function. Differential gene expression analysis was then performed with the exactTest function. Pairwise comparison was conducted between mock samples and infected samples at different time points. Resultant gene-wise log fold change (log FC), *P* value, and FDR were utilized in subsequent analyses.

### (vii) TE analysis.

The calculation of translational efficiency involved normalizing counts to account for library size in edgeR to generate log_2_ CPM estimates for each gene in ribo-seq and RNA-seq and subtracting log_2_ CPM in RNA-seq from log_2_ CPM in ribo-seq to provide an estimate of the difference in expression level between ribo-seq and RNA-seq for a given gene (the TE value). Mean mock TE was subtracted from mean infected TE to calculate ΔTE.

### (viii) Clustered time course expression analysis.

Clustered time course expression analysis, cluster profile plots, and gene set overrepresentation analysis were adapted from the work of Puray-Chavez et al. (83). Heat maps were generated with the ComplexHeatmap R package (84). In brief, for RNA-seq and ribo-seq experiments with HBEC and Vero cells, count data were filtered for genes with an FDR of <0.05 and a |log FC| of >1 in at least one time point in the DE analysis. RNA-seq and ribo-seq log CPM values of each time point were each converted to per-gene z-scores. Consensus clustering was performed with the ConsensusClusteringPlus R package (85) using the following nondefault parameters: maxK = 13, reps = 100, innerLinkage = “complete,” and finalLinkage = “ward.D2.” Cluster merging and reordering were conducted based on manual inspection. Clusters with immune response pathways as determined by the overrepresentation analysis were labeled on the heat map, along with relevant ISGs. Log FC of differentially expressed genes from DE analysis were plotted across the time course grouped by clusters, along with mean log FC of each time point.

### (ix) Gene set enrichment analysis.

Overrepresentation of gene sets in each cluster was analyzed using the enricher function of the clusterProfiler R package (86). Gene sets were retrieved from the Molecular Signature Database with the msigdbr R package, including “Hallmark” and “GO:BP” (87–90). Gene sets were considered significantly overrepresented if adjusted *P* values (*q* values) were below 0.1 and were selected manually for plotting.

### Viral counts.

Viral read density plots were generated using the SAM file from viral genome alignment. The SAMtools (79) package was used to create an mpileup file containing information about the read density, strandedness, mapping quality, and nucleotide identity at each position. Custom scripts (deposited at GitHub under kutluaylab) then were utilized to create files providing only the nucleotide identity and number of counts at each position for both sense and antisense reads. These were then visualized by scripts written in R.

As SARS-CoV-2 generates chimeric subgenomic mRNAs (sgRNAs) in addition to its genomic RNA (gRNA), featureCounts could not be used to accurately estimate viral gene counts from RNA-seq due to the presence of nested 3′ sequences. Therefore, in order to visualize and enumerate such chimeric sequences, the Burrows-Wheeler aligner (BWA) (91) was used in “mem” mode on viral RNA-seq reads. After this alignment was generated using the default parameters and the reference SARS-CoV-2 FASTA file mentioned above, chimeric reads were isolated by searching for all reads containing the SA tag and the SARS-CoV-2 transcriptional regulatory sequence (TRS) AAACGAAC. SARS-CoV-2 gRNAs were extracted by searching for all reads containing the first 15 to 20 bases of the ORF1A CDS, as these sequences would be present only in full-length SARS-CoV-2 genomes. This provided the sequences and alignment locations of the chimeric and genomic reads, which were then visualized using R. For sgRNAs, the viral gene corresponding to each transcript was determined by locating the CDS with the nearest downstream start site. These data, together with the number of gRNAs, were used to calculate relative percentages of viral transcripts and, together with the total number of mapped viral reads, allowed the tabulation of viral gene counts at each time point. For ribosome profiling data, featureCounts was used to enumerate viral reads, as ribosomes translate only the first gene on each transcript, and so footprints from nested 3′ genes were low enough to be negligible.

### Data availability.

All ribo-seq and RNA-seq data were deposited in the GEO database under accession number GSE158930.

## References

[1] Masters PS. 2006. The molecular biology of coronaviruses. Adv Virus Res 66:193–292. doi:10.1016/S0065-3527(06)66005-3. 16877062 PMC7112330

[2] Weiss SR, Navas-Martin S. 2005. Coronavirus pathogenesis and the emerging pathogen severe acute respiratory syndrome coronavirus. Microbiol Mol Biol Rev 69:635–664. doi:10.1128/MMBR.69.4.635-664.2005. 16339739 PMC1306801

[3] Sa Ribero M, Jouvenet N, Dreux M, Nisole S. 2020. Interplay between SARS-CoV-2 and the type I interferon response. PLoS Pathog 16:e1008737. doi:10.1371/journal.ppat.1008737. 32726355 PMC7390284

[4] Blanco-Melo D, Nilsson-Payant BE, Liu W-C, Uhl S, Hoagland D, Møller R, Jordan TX, Oishi K, Panis M, Sachs D, Wang TT, Schwartz RE, Lim JK, Albrecht RA, tenOever BR. 2020. Imbalanced host response to SARS-CoV-2 drives development of COVID-19. Cell 181:1036–1045.E1039. doi:10.1016/j.cell.2020.04.026. 32416070 PMC7227586

[5] Zhang X, Tan Y, Ling Y, Lu G, Liu F, Yi Z, Jia X, Wu M, Shi B, Xu S, Chen J, Wang W, Chen B, Jiang L, Yu S, Lu J, Wang J, Xu M, Yuan Z, Zhang Q, Zhang X, Zhao G, Wang S, Chen S, Lu H. 2020. Viral and host factors related to the clinical outcome of COVID-19. Nature 583:437–440. doi:10.1038/s41586-020-2355-0. 32434211

[6] Lokugamage KG, Hage A, de Vries M, Valero-Jimenez AM, Schindewolf C, Dittmann M, Rajsbaum R, Menachery VD. 2020. Type I interferon susceptibility distinguishes SARS-CoV-2 from SARS-CoV. J Virol 94:e01410-20. doi:10.1128/JVI.01410-20. PMC7654262 32938761

[7] Schubert K, Karousis ED, Jomaa A, Scaiola A, Echeverria B, Gurzeler L-A, Leibundgut M, Thiel V, Mühlemann O, Ban N. 2020. SARS-CoV-2 Nsp1 binds the ribosomal mRNA channel to inhibit translation. Nat Struct Mol Biol 27:959–966. doi:10.1038/s41594-020-0511-8. 32908316

[8] Banerjee AK, Blanco MR, Bruce EA, Honson DD, Chen LM, Chow A, Bhat P, Ollikainen N, Quinodoz SA, Loney C, Thai J, Miller ZD, Lin AE, Schmidt MM, Stewart DG, Goldfarb D, De Lorenzo G, Rihn SJ, Voorhees RM, Botten JW, Majumdar D, Guttman M. 2020. SARS-CoV-2 disrupts splicing, translation, and protein trafficking to suppress host defenses. Cell 183:1325–1339.E1321. doi:10.1016/j.cell.2020.10.004. 33080218 PMC7543886

[9] Thoms M, Buschauer R, Ameismeier M, Koepke L, Denk T, Hirschenberger M, Kratzat H, Hayn M, Mackens-Kiani T, Cheng J, Straub JH, Stürzel CM, Fröhlich T, Berninghausen O, Becker T, Kirchhoff F, Sparrer KMJ, Beckmann R. 2020. Structural basis for translational shutdown and immune evasion by the Nsp1 protein of SARS-CoV-2. Science 369:1249–1255. doi:10.1126/science.abc8665. 32680882 PMC7402621

[10] Lapointe CP, Grosely R, Johnson AG, Wang J, Fernandez IS, Puglisi JD. 2021. Dynamic competition between SARS-CoV-2 NSP1 and mRNA on the human ribosome inhibits translation initiation. Proc Natl Acad Sci USA 118:e2017715118. doi:10.1073/pnas.2017715118. 33479166 PMC8017934

[11] Hsu JC, Laurent-Rolle M, Pawlak JB, Wilen CB, Cresswell P. 2021. Translational shutdown and evasion of the innate immune response by SARS-CoV-2 NSP14 protein. Proc Natl Acad Sci USA 118:e2101161118. doi:10.1073/pnas.2101161118. 34045361 PMC8214666

[12] Tidu A, Janvier A, Schaeffer L, Sosnowski P, Kuhn L, Hammann P, Westhof E, Eriani G, Martin F. 2020. The viral protein NSP1 acts as a ribosome gatekeeper for shutting down host translation and fostering SARS-CoV-2 translation. RNA 594:253–264. doi:10.1261/rna.078121.120. PMC7901841 33268501

[13] Finkel Y, Gluck A, Nachshon A, Winkler R, Fisher T, Rozman B, Mizrahi O, Lubelsky Y, Zuckerman B, Slobodin B, Yahalom-Ronen Y, Tamir H, Ulitsky I, Israely T, Paran N, Schwartz M, Stern-Ginossar N. 2021. SARS-CoV-2 uses a multipronged strategy to impede host protein synthesis. Nature 594:240–245. doi:10.1038/s41586-021-03610-3. 33979833

[14] Butler DJ, Mozsary C, Meydan C, Danko D, Foox J, Rosiene J, Shaiber A, Afshinnekoo E, MacKay M, Sedlazeck FJ, Ivanov NA, Sierra M, Pohle D, Zietz M, Gisladottir U, Ramlall V, Westover CD, Ryon K, Young B, Bhattacharya C, Ruggiero P, Langhorst BW, Tanner N, Gawrys J, Meleshko D, Xu D, Steel PAD, Shemesh AJ, Xiang J, Thierry-Mieg J, Thierry-Mieg D, Schwartz RE, Iftner A, Bezdan D, Sipley J, Cong L, Craney A, Velu P, Melnick AM, Hajirasouliha I, Horner SM, Iftner T, Salvatore M, Loda M, Westblade LF, Cushing M, Levy S, Wu S, Tatonetti N, Imielinski M, Rennert H, Mason CE. 2020. Shotgun transcriptome and isothermal profiling of SARS-CoV-2 infection reveals unique host responses, viral diversification, and drug interactions. bioRxiv doi:10.1101/2020.04.20.048066. PMC7954844 33712587

[15] Menachery VD, Eisfeld AJ, Schäfer A, Josset L, Sims AC, Proll S, Fan S, Li C, Neumann G, Tilton SC, Chang J, Gralinski LE, Long C, Green R, Williams CM, Weiss J, Matzke MM, Webb-Robertson B-J, Schepmoes AA, Shukla AK, Metz TO, Smith RD, Waters KM, Katze MG, Kawaoka Y, Baric RS. 2014. Pathogenic influenza viruses and coronaviruses utilize similar and contrasting approaches to control interferon-stimulated gene responses. mBio 5:e01174-14. doi:10.1128/mBio.01174-14. 24846384 PMC4030454

[16] Mitchell HD, Eisfeld AJ, Sims AC, McDermott JE, Matzke MM, Webb-Robertson B-JM, Tilton SC, Tchitchek N, Josset L, Li C, Ellis AL, Chang JH, Heegel RA, Luna ML, Schepmoes AA, Shukla AK, Metz TO, Neumann G, Benecke AG, Smith RD, Baric RS, Kawaoka Y, Katze MG, Waters KM. 2013. A network integration approach to predict conserved regulators related to pathogenicity of influenza and SARS-CoV respiratory viruses. PLoS One 8:e69374. doi:10.1371/journal.pone.0069374. 23935999 PMC3723910

[17] Wilk AJ, Rustagi A, Zhao NQ, Roque J, Martínez-Colón GJ, McKechnie JL, Ivison GT, Ranganath T, Vergara R, Hollis T, Simpson LJ, Grant P, Subramanian A, Rogers AJ, Blish CA. 2020. A single-cell atlas of the peripheral immune response in patients with severe COVID-19. Nat Med 26:1070–1076. doi:10.1038/s41591-020-0944-y. 32514174 PMC7382903

[18] Zhou Z, Ren L, Zhang L, Zhong J, Xiao Y, Jia Z, Guo L, Yang J, Wang C, Jiang S, Yang D, Zhang G, Li H, Chen F, Xu Y, Chen M, Gao Z, Yang J, Dong J, Liu B, Zhang X, Wang W, He K, Jin Q, Li M, Wang J. 2020. Heightened innate immune responses in the respiratory tract of COVID-19 patients. Cell Host Microbe 27:883–890.E882. doi:10.1016/j.chom.2020.04.017. 32407669 PMC7196896

[19] Alexander MR, Brice AM, van Vuren PJ, Rootes CL, Tribolet L, Cowled C, Bean AGD, Stewart CR. 2021. Ribosome-profiling reveals restricted post transcriptional expression of antiviral cytokines and transcription factors during SARS-CoV-2 infection. Int J Mol Sci 22:3392. doi:10.3390/ijms22073392. 33806254 PMC8036502

[20] Finkel Y, Mizrahi O, Nachshon A, Weingarten-Gabbay S, Morgenstern D, Yahalom-Ronen Y, Tamir H, Achdout H, Stein D, Israeli O, Beth-Din A, Melamed S, Weiss S, Israely T, Paran N, Schwartz M, Stern-Ginossar N. 2021. The coding capacity of SARS-CoV-2. Nature 589:125–130. doi:10.1038/s41586-020-2739-1. 32906143

[21] Nakagawa K, Lokugamage KG, Makino S. 2016. Viral and cellular mRNA translation in coronavirus-infected cells. Adv Virus Res 96:165–192. doi:10.1016/bs.aivir.2016.08.001. 27712623 PMC5388242

[22] Plant EP, Dinman JD. 2008. The role of programmed-1 ribosomal frameshifting in coronavirus propagation. Front Biosci 13:4873–4881. doi:10.2741/3046. 18508552 PMC2435135

[23] Plant EP, Jacobs KLM, Harger JW, Meskauskas A, Jacobs JL, Baxter JL, Petrov AN, Dinman JD. 2003. The 9-A solution: how mRNA pseudoknots promote efficient programmed -1 ribosomal frameshifting. RNA 9:168–174. doi:10.1261/rna.2132503. 12554858 PMC1237042

[24] Korniy N, Samatova E, Anokhina MM, Peske F, Rodnina MV. 2019. Mechanisms and biomedical implications of -1 programmed ribosome frameshifting on viral and bacterial mRNAs. FEBS Lett 593:1468–1482. doi:10.1002/1873-3468.13478. 31222875 PMC6771820

[25] Irigoyen N, Firth AE, Jones JD, Chung BY-W, Siddell SG, Brierley I. 2016. High-resolution analysis of coronavirus gene expression by RNA sequencing and ribosome profiling. PLoS Pathog 12:e1005473. doi:10.1371/journal.ppat.1005473. 26919232 PMC4769073

[26] Baril M, Dulude D, Gendron K, Lemay G, Brakier-Gingras L. 2003. Efficiency of a programmed -1 ribosomal frameshift in the different subtypes of the human immunodeficiency virus type 1 group M. RNA 9:1246–1253. doi:10.1261/rna.5113603. 13130138 PMC1370488

[27] Dulude D, Berchiche YA, Gendron K, Brakier-Gingras L, Heveker N. 2006. Decreasing the frameshift efficiency translates into an equivalent reduction of the replication of the human immunodeficiency virus type 1. Virology 345:127–136. doi:10.1016/j.virol.2005.08.048. 16256163

[28] Jacks T, Power MD, Masiarz FR, Luciw PA, Barr PJ, Varmus HE. 1988. Characterization of ribosomal frameshifting in HIV-1 gag-pol expression. Nature 331:280–283. doi:10.1038/331280a0. 2447506

[29] Lan TCT, Allan MF, Malsick LE, Woo JZ, Zhu C, Zhang F, Khandwala S, Nyeo SSY, Sun Y, Guo JU, Bathe M, Näär A, Griffiths A, Rouskin S. 2022. Secondary structural ensembles of the SARS-CoV-2 RNA genome in infected cells. Nat Commun 13:1128. doi:10.1038/s41467-022-28603-2. 35236847 PMC8891300

[30] Ingolia NT, Brar GA, Rouskin S, McGeachy AM, Weissman JS. 2012. The ribosome profiling strategy for monitoring translation in vivo by deep sequencing of ribosome-protected mRNA fragments. Nat Protoc 7:1534–1550. doi:10.1038/nprot.2012.086. 22836135 PMC3535016

[31] Ingolia NT, Ghaemmaghami S, Newman JR, Weissman JS. 2009. Genome-wide analysis in vivo of translation with nucleotide resolution using ribosome profiling. Science 324:218–223. doi:10.1126/science.1168978. 19213877 PMC2746483

[32] Kim D, Lee J-Y, Yang J-S, Kim JW, Kim VN, Chang H. 2020. The architecture of SARS-CoV-2 transcriptome. Cell 181:914–921.E910. doi:10.1016/j.cell.2020.04.011. 32330414 PMC7179501

[33] Huang J, Hume AJ, Abo KM, Werder RB, Villacorta-Martin C, Alysandratos KD, Beermann ML, Simone-Roach C, Lindstrom-Vautrin J, Olejnik J, Suder EL, Bullitt E, Hinds A, Sharma A, Bosmann M, Wang R, Hawkins F, Burks EJ, Saeed M, Wilson AA, Muhlberger E, Kotton DN. 2020. SARS-CoV-2 infection of pluripotent stem cell-derived human lung alveolar type 2 cells elicits a rapid epithelial-intrinsic inflammatory response. bioRxiv doi:10.1101/2020.06.30.175695. PMC7500949 32979316

[34] Dinan AM, Keep S, Bickerton E, Britton P, Firth AE, Brierley I. 2019. Comparative analysis of gene expression in virulent and attenuated strains of infectious bronchitis virus at subcodon resolution. J Virol 93:e00714-19. doi:10.1128/JVI.00714-19. 31243124 PMC6714804

[35] Schaefer I-M, Padera RF, Solomon IH, Kanjilal S, Hammer MM, Hornick JL, Sholl LM. 2020. In situ detection of SARS-CoV-2 in lungs and airways of patients with COVID-19. Mod Pathol 33:2104–2114. doi:10.1038/s41379-020-0595-z. 32561849 PMC7304376

[36] Kwan T, Thompson SR. 2019. Noncanonical translation initiation in eukaryotes. Cold Spring Harb Perspect Biol 11:a032672. doi:10.1101/cshperspect.a032672. 29959190 PMC6442200

[37] James CC, Smyth JW. 2018. Alternative mechanisms of translation initiation: an emerging dynamic regulator of the proteome in health and disease. Life Sci 212:138–144. doi:10.1016/j.lfs.2018.09.054. 30290184 PMC6345546

[38] Lei X, Dong X, Ma R, Wang W, Xiao X, Tian Z, Wang C, Wang Y, Li L, Ren L, Guo F, Zhao Z, Zhou Z, Xiang Z, Wang J. 2020. Activation and evasion of type I interferon responses by SARS-CoV-2. Nat Commun 11:3810. doi:10.1038/s41467-020-17665-9. 32733001 PMC7392898

[39] Xia H, Cao Z, Xie X, Zhang X, Chen JY-C, Wang H, Menachery VD, Rajsbaum R, Shi P-Y. 2020. Evasion of type I interferon by SARS-CoV-2. Cell Rep 33:108234. doi:10.1016/j.celrep.2020.108234. 32979938 PMC7501843

[40] Farabaugh PJ. 1996. Programmed translational frameshifting. Microbiol Rev 60:103–134. doi:10.1128/mr.60.1.103-134.1996. 8852897 PMC239420

[41] Dinman JD. 2012. Mechanisms and implications of programmed translational frameshifting. Wiley Interdiscip Rev RNA 3:661–673. doi:10.1002/wrna.1126. 22715123 PMC3419312

[42] Bhatt PR, Scaiola A, Loughran G, Leibundgut M, Kratzel A, Meurs R, Dreos R, O'Connor KM, McMillan A, Bode JW, Thiel V, Gatfield D, Atkins JF, Ban N. 2021. Structural basis of ribosomal frameshifting during translation of the SARS-CoV-2 RNA genome. Science 372:1306–1313. doi:10.1126/science.abf3546. 34029205 PMC8168617

[43] Giedroc DP, Cornish PV. 2009. Frameshifting RNA pseudoknots: structure and mechanism. Virus Res 139:193–208. doi:10.1016/j.virusres.2008.06.008. 18621088 PMC2670756

[44] Wang X, Xuan Y, Han Y, Ding X, Ye K, Yang F, Gao P, Goff SP, Gao G. 2019. Regulation of HIV-1 Gag-Pol expression by Shiftless, an inhibitor of programmed -1 ribosomal frameshifting. Cell 176:625–635.E614. doi:10.1016/j.cell.2018.12.030. 30682371 PMC8486322

[45] Yu D, Zhao Y, Pan J, Yang X, Liang Z, Xie S, Cao R. 2021. C19orf66 inhibits Japanese encephalitis virus replication by targeting -1 PRF and the NS3 protein. Virol Sin 36:1443–1455. doi:10.1007/s12250-021-00423-6. 34309824 PMC8692527

[46] Suzuki Y, Chin W-X, Han QEn, Ichiyama K, Lee CH, Eyo ZW, Ebina H, Takahashi H, Takahashi C, Tan BH, Hishiki T, Ohba K, Matsuyama T, Koyanagi Y, Tan Y-J, Sawasaki T, Chu JJH, Vasudevan SG, Sano K, Yamamoto N. 2016. Characterization of RyDEN (C19orf66) as an interferon-stimulated cellular inhibitor against dengue virus replication. PLoS Pathog 12:e1005357. doi:10.1371/journal.ppat.1005357. 26735137 PMC4703206

[47] Napthine S, Hill CH, Nugent HCM, Brierley I. 2021. Modulation of viral programmed ribosomal frameshifting and stop codon readthrough by the host restriction factor Shiftless. Viruses 13:1230. doi:10.3390/v13071230. 34202160 PMC8310280

[48] Schmidt N, Lareau CA, Keshishian H, Ganskih S, Schneider C, Hennig T, Melanson R, Werner S, Wei Y, Zimmer M, Ade J, Kirschner L, Zielinski S, Dölken L, Lander ES, Caliskan N, Fischer U, Vogel J, Carr SA, Bodem J, Munschauer M. 2021. The SARS-CoV-2 RNA-protein interactome in infected human cells. Nat Microbiol 6:339–353. doi:10.1038/s41564-020-00846-z. 33349665 PMC7906908

[49] Ravindra NG, Alfajaro MM, Gasque V, Huston NC, Wan H, Szigeti-Buck K, Yasumoto Y, Greaney AM, Habet V, Chow RD, Chen JS, Wei J, Filler RB, Wang B, Wang G, Niklason LE, Montgomery RR, Eisenbarth SC, Chen S, Williams A, Iwasaki A, Horvath TL, Foxman EF, Pierce RW, Pyle AM, van Dijk D, Wilen CB. 2021. Single-cell longitudinal analysis of SARS-CoV-2 infection in human airway epithelium identifies target cells, alterations in gene expression, and cell state changes. PLoS Biol 19:e3001143. doi:10.1371/journal.pbio.3001143. 33730024 PMC8007021

[50] Rao S, Hoskins I, Tonn T, Garcia D, Ozadam H, Cenik ES, Cenik C. 2021. Genes with 5′ terminal oligopyrimidine tracts preferentially escape global suppression of translation by the SARS-CoV-2 Nsp1 protein. bioRxiv doi:10.1101/2020.09.13.295493. PMC8370740 34127534

[51] Konno Y, Kimura I, Uriu K, Fukushi M, Irie T, Koyanagi Y, Sauter D, Gifford RJ, Nakagawa S, Sato K, USFQ-COVID19 Consortium. 2020. SARS-CoV-2 ORF3b is a potent interferon antagonist whose activity is increased by a naturally occurring elongation variant. Cell Rep 32:108185. doi:10.1016/j.celrep.2020.108185. 32941788 PMC7473339

[52] Miorin L, Kehrer T, Sanchez-Aparicio MT, Zhang K, Cohen P, Patel RS, Cupic A, Makio T, Mei M, Moreno E, Danziger O, White KM, Rathnasinghe R, Uccellini M, Gao S, Aydillo T, Mena I, Yin X, Martin-Sancho L, Krogan NJ, Chanda SK, Schotsaert M, Wozniak RW, Ren Y, Rosenberg BR, Fontoura BMA, García-Sastre A. 2020. SARS-CoV-2 Orf6 hijacks Nup98 to block STAT nuclear import and antagonize interferon signaling. Proc Natl Acad Sci USA 117:28344–28354. doi:10.1073/pnas.2016650117. 33097660 PMC7668094

[53] Setaro AC, Gaglia MM. 2021. All hands on deck: SARS-CoV-2 proteins that block early anti-viral interferon responses. Curr Res Virol Sci 2:100015. doi:10.1016/j.crviro.2021.100015. 34786565 PMC8588586

[54] Hayn M, Hirschenberger M, Koepke L, Nchioua R, Straub JH, Klute S, Hunszinger V, Zech F, Prelli Bozzo C, Aftab W, Christensen MH, Conzelmann C, Müller JA, Srinivasachar Badarinarayan S, Stürzel CM, Forne I, Stenger S, Conzelmann K-K, Münch J, Schmidt FI, Sauter D, Imhof A, Kirchhoff F, Sparrer KMJ. 2021. Systematic functional analysis of SARS-CoV-2 proteins uncovers viral innate immune antagonists and remaining vulnerabilities. Cell Rep 35:109126. doi:10.1016/j.celrep.2021.109126. 33974846 PMC8078906

[55] Li J-Y, Liao C-H, Wang Q, Tan Y-J, Luo R, Qiu Y, Ge X-Y. 2020. The ORF6, ORF8 and nucleocapsid proteins of SARS-CoV-2 inhibit type I interferon signaling pathway. Virus Res 286:198074. doi:10.1016/j.virusres.2020.198074. 32589897 PMC7309931

[56] Shemesh M, Aktepe TE, Deerain JM, McAuley JL, Audsley MD, David CT, Purcell DFJ, Urin V, Hartmann R, Moseley GW, Mackenzie JM, Schreiber G, Harari D. 2021. SARS-CoV-2 suppresses IFNbeta production mediated by NSP1, 5, 6, 15, ORF6 and ORF7b but does not suppress the effects of added interferon. PLoS Pathog 17:e1009800. doi:10.1371/journal.ppat.1009800. 34437657 PMC8389490

[57] Hyde JL, Diamond MS. 2015. Innate immune restriction and antagonism of viral RNA lacking 2-O methylation. Virology 479–480:66–74. doi:10.1016/j.virol.2015.01.019. PMC4424151 25682435

[58] Fensterl V, Sen GC. 2015. Interferon-induced Ifit proteins: their role in viral pathogenesis. J Virol 89:2462–2468. doi:10.1128/JVI.02744-14. 25428874 PMC4325746

[59] Reynaud JM, Kim DY, Atasheva S, Rasalouskaya A, White JP, Diamond MS, Weaver SC, Frolova EI, Frolov I. 2015. IFIT1 differentially interferes with translation and replication of alphavirus genomes and promotes induction of type I interferon. PLoS Pathog 11:e1004863. doi:10.1371/journal.ppat.1004863. 25927359 PMC4415776

[60] Daffis S, Szretter KJ, Schriewer J, Li J, Youn S, Errett J, Lin T-Y, Schneller S, Zust R, Dong H, Thiel V, Sen GC, Fensterl V, Klimstra WB, Pierson TC, Buller RM, Gale M, Shi P-Y, Diamond MS. 2010. 2'-O methylation of the viral mRNA cap evades host restriction by IFIT family members. Nature 468:452–456. doi:10.1038/nature09489. 21085181 PMC3058805

[61] Diamond MS, Farzan M. 2013. The broad-spectrum antiviral functions of IFIT and IFITM proteins. Nat Rev Immunol 13:46–57. doi:10.1038/nri3344. 23237964 PMC3773942

[62] Burke JM, St Clair LA, Perera R, Parker R. 2021. SARS-CoV-2 infection triggers widespread host mRNA decay leading to an mRNA export block. RNA 27:1318–1329. doi:10.1261/rna.078923.121. 34315815 PMC8522697

[63] Zhang K, Miorin L, Makio T, Dehghan I, Gao S, Xie Y, Zhong H, Esparza M, Kehrer T, Kumar A, Hobman TC, Ptak C, Gao B, Minna JD, Chen Z, Garcia-Sastre A, Ren Y, Wozniak RW, Fontoura BMA. 2021. Nsp1 protein of SARS-CoV-2 disrupts the mRNA export machinery to inhibit host gene expression. Sci Adv 7:eabe7386. doi:10.1126/sciadv.abe7386. 33547084 PMC7864571

[64] Lovell D, Pawlowsky-Glahn V, Egozcue JJ, Marguerat S, Bahler J. 2015. Proportionality: a valid alternative to correlation for relative data. PLoS Comput Biol 11:e1004075. doi:10.1371/journal.pcbi.1004075. 25775355 PMC4361748

[65] Morton JT, Marotz C, Washburne A, Silverman J, Zaramela LS, Edlund A, Zengler K, Knight R. 2019. Establishing microbial composition measurement standards with reference frames. Nat Commun 10:2719. doi:10.1038/s41467-019-10656-5. 31222023 PMC6586903

[66] Channappanavar R, Perlman S. 2017. Pathogenic human coronavirus infections: causes and consequences of cytokine storm and immunopathology. Semin Immunopathol 39:529–539. doi:10.1007/s00281-017-0629-x. 28466096 PMC7079893

[67] Perlman S, Dandekar AA. 2005. Immunopathogenesis of coronavirus infections: implications for SARS. Nat Rev Immunol 5:917–927. doi:10.1038/nri1732. 16322745 PMC7097326

[68] Jafarzadeh A, Chauhan P, Saha B, Jafarzadeh S, Nemati M. 2020. Contribution of monocytes and macrophages to the local tissue inflammation and cytokine storm in COVID-19: lessons from SARS and MERS, and potential therapeutic interventions. Life Sci 257:118102. doi:10.1016/j.lfs.2020.118102. 32687918 PMC7367812

[69] Xie X, Muruato A, Lokugamage KG, Narayanan K, Zhang X, Zou J, Liu J, Schindewolf C, Bopp NE, Aguilar PV, Plante KS, Weaver SC, Makino S, LeDuc JW, Menachery VD, Shi P-Y. 2020. An infectious cDNA clone of SARS-CoV-2. Cell Host Microbe 27:841–848.E843. doi:10.1016/j.chom.2020.04.004. 32289263 PMC7153529

[70] Case JB, Rothlauf PW, Chen RE, Liu Z, Zhao H, Kim AS, Bloyet L-M, Zeng Q, Tahan S, Droit L, Ilagan MXG, Tartell MA, Amarasinghe G, Henderson JP, Miersch S, Ustav M, Sidhu S, Virgin HW, Wang D, Ding S, Corti D, Theel ES, Fremont DH, Diamond MS, Whelan SPJ. 2020. Neutralizing antibody and soluble ACE2 inhibition of a replication-competent VSV-SARS-CoV-2 and a clinical isolate of SARS-CoV-2. Cell Host Microbe 28:475–485.E475. doi:10.1016/j.chom.2020.06.021. 32735849 PMC7332453

[71] Horani A, Druley TE, Zariwala MA, Patel AC, Levinson BT, Van Arendonk LG, Thornton KC, Giacalone JC, Albee AJ, Wilson KS, Turner EH, Nickerson DA, Shendure J, Bayly PV, Leigh MW, Knowles MR, Brody SL, Dutcher SK, Ferkol TW. 2012. Whole-exome capture and sequencing identifies HEATR2 mutation as a cause of primary ciliary dyskinesia. Am J Hum Genet 91:685–693. doi:10.1016/j.ajhg.2012.08.022. 23040496 PMC3484505

[72] You Y, Richer EJ, Huang T, Brody SL. 2002. Growth and differentiation of mouse tracheal epithelial cells: selection of a proliferative population. Am J Physiol Lung Cell Mol Physiol 283:L1315–1321. doi:10.1152/ajplung.00169.2002. 12388377

[73] Horani A, Ustione A, Huang T, Firth AL, Pan J, Gunsten SP, Haspel JA, Piston DW, Brody SL. 2018. Establishment of the early cilia preassembly protein complex during motile ciliogenesis. Proc Natl Acad Sci USA 115:E1221–E1228. doi:10.1073/pnas.1715915115. 29358401 PMC5819421

[74] Kutluay SB, Zang T, Blanco-Melo D, Powell C, Jannain D, Errando M, Bieniasz PD. 2014. Global changes in the RNA binding specificity of HIV-1 gag regulate virion genesis. Cell 159:1096–1109. doi:10.1016/j.cell.2014.09.057. 25416948 PMC4247003

[75] Kutluay SB, Bieniasz PD. 2016. Analysis of HIV-1 Gag-RNA interactions in cells and virions by CLIP-seq. Methods Mol Biol 1354:119–131. doi:10.1007/978-1-4939-3046-3_8. 26714708 PMC6548315

[76] Langmead B, Trapnell C, Pop M, Salzberg SL. 2009. Ultrafast and memory-efficient alignment of short DNA sequences to the human genome. Genome Biol 10:R25. doi:10.1186/gb-2009-10-3-r25. 19261174 PMC2690996

[77] Dobin A, Davis CA, Schlesinger F, Drenkow J, Zaleski C, Jha S, Batut P, Chaisson M, Gingeras TR. 2013. STAR: ultrafast universal RNA-seq aligner. Bioinformatics 29:15–21. doi:10.1093/bioinformatics/bts635. 23104886 PMC3530905

[78] Liao Y, Smyth GK, Shi W. 2014. featureCounts: an efficient general purpose program for assigning sequence reads to genomic features. Bioinformatics 30:923–930. doi:10.1093/bioinformatics/btt656. 24227677

[79] Li H, Handsaker B, Wysoker A, Fennell T, Ruan J, Homer N, Marth G, Abecasis G, Durbin R, 1000 Genome Project Data Processing Subgroup. 2009. The Sequence Alignment/Map format and SAMtools. Bioinformatics 25:2078–2079. doi:10.1093/bioinformatics/btp352. 19505943 PMC2723002

[80] Robinson MD, McCarthy DJ, Smyth GK. 2010. edgeR: a Bioconductor package for differential expression analysis of digital gene expression data. Bioinformatics 26:139–140. doi:10.1093/bioinformatics/btp616. 19910308 PMC2796818

[81] Lauria F, Tebaldi T, Bernabò P, Groen EJN, Gillingwater TH, Viero G. 2018. riboWaltz: optimization of ribosome P-site positioning in ribosome profiling data. PLoS Comput Biol 14:e1006169. doi:10.1371/journal.pcbi.1006169. 30102689 PMC6112680

[82] Zhang P, He D, Xu Y, Hou J, Pan B-F, Wang Y, Liu T, Davis CM, Ehli EA, Tan L, Zhou F, Hu J, Yu Y, Chen X, Nguyen TM, Rosen JM, Hawke DH, Ji Z, Chen Y. 2017. Genome-wide identification and differential analysis of translational initiation. Nat Commun 8:1749. doi:10.1038/s41467-017-01981-8. 29170441 PMC5701008

[83] Puray-Chavez M, LaPak KM, Schrank TP, Elliott JL, Bhatt DP, Agajanian MJ, Jasuja R, Lawson DQ, Davis K, Rothlauf PW, Liu Z, Jo H, Lee N, Tenneti K, Eschbach JE, Shema Mugisha C, Cousins EM, Cloer EW, Vuong HR, VanBlargan LA, Bailey AL, Gilchuk P, Crowe JE, Diamond MS, Hayes DN, Whelan SPJ, Horani A, Brody SL, Goldfarb D, Major MB, Kutluay SB. 2021. Systematic analysis of SARS-CoV-2 infection of an ACE2-negative human airway cell. Cell Rep 36:109364. doi:10.1016/j.celrep.2021.109364. 34214467 PMC8220945

[84] Gu Z, Eils R, Schlesner M. 2016. Complex heatmaps reveal patterns and correlations in multidimensional genomic data. Bioinformatics 32:2847–2849. doi:10.1093/bioinformatics/btw313. 27207943

[85] Wilkerson MD, Hayes DN. 2010. ConsensusClusterPlus: a class discovery tool with confidence assessments and item tracking. Bioinformatics 26:1572–1573. doi:10.1093/bioinformatics/btq170. 20427518 PMC2881355

[86] Yu G, Wang LG, Han Y, He QY. 2012. clusterProfiler: an R package for comparing biological themes among gene clusters. OMICS 16:284–287. doi:10.1089/omi.2011.0118. 22455463 PMC3339379

[87] Liberzon A, Birger C, Thorvaldsdóttir H, Ghandi M, Mesirov JP, Tamayo P. 2015. The Molecular Signatures Database (MSigDB) hallmark gene set collection. Cell Syst 1:417–425. doi:10.1016/j.cels.2015.12.004. 26771021 PMC4707969

[88] Ashburner M, Ball CA, Blake JA, Botstein D, Butler H, Cherry JM, Davis AP, Dolinski K, Dwight SS, Eppig JT, Harris MA, Hill DP, Issel-Tarver L, Kasarskis A, Lewis S, Matese JC, Richardson JE, Ringwald M, Rubin GM, Sherlock G. 2000. Gene ontology: tool for the unification of biology. The Gene Ontology Consortium. Nat Genet 25:25–29. doi:10.1038/75556. 10802651 PMC3037419

[89] Liberzon A. 2014. A description of the Molecular Signatures Database (MSigDB) web site. Methods Mol Biol 1150:153–160. doi:10.1007/978-1-4939-0512-6_9. 24743996

[90] Liberzon A, Subramanian A, Pinchback R, Thorvaldsdóttir H, Tamayo P, Mesirov JP. 2011. Molecular signatures database (MSigDB) 3.0. Bioinformatics 27:1739–1740. doi:10.1093/bioinformatics/btr260. 21546393 PMC3106198

[91] Li H, Durbin R. 2009. Fast and accurate short read alignment with Burrows-Wheeler transform. Bioinformatics 25:1754–1760. doi:10.1093/bioinformatics/btp324. 19451168 PMC2705234

